# A systematic review and meta-analysis uncovering the relationship between alcohol consumption and sickness absence. When type of design, data, and sickness absence make a difference

**DOI:** 10.1371/journal.pone.0262458

**Published:** 2022-01-11

**Authors:** Neda S. Hashemi, Jens Christoffer Skogen, Aleksandra Sevic, Mikkel Magnus Thørrisen, Silje Lill Rimstad, Hildegunn Sagvaag, Heleen Riper, Randi Wågø Aas

**Affiliations:** 1 Department of Public Health, Faculty of Health Sciences, University of Stavanger, Stavanger, Norway; 2 Department of Health Promotion, Norwegian Institute of Public Health, Bergen, Norway; 3 Alcohol & Drug Research Western Norway, Stavanger University Hospital, Stavanger, Norway; 4 Department of Occupational Therapy, Prosthetics and Orthotics, Faculty of Health Sciences, OsloMet – Oslo Metropolitan University, Oslo, Norway; 5 West Norway Competence Centre (KoRus Stavanger)/Rogaland A-Centre, Stavanger, Norway; 6 Department of Clinical, Neuro, & Developmental Psychology, Faculty of Behavioral and Movement Sciences, VU Amsterdam, Amsterdam, Netherlands; 7 Department of Psychiatry, Amsterdam Public Health Research Institute, VU University Medical Center, Amsterdam, Netherlands; 8 Department of Clinical Research, Research Unit for Telepsychiatry and e-Mental Health, University of Southern Denmark, Odense, Denmark; 9 Research Centre for Child Psychiatry, University of Turku, Turku, Finland; University of Queensland, AUSTRALIA

## Abstract

**Aim:**

Earlier research has revealed a strong relationship between alcohol use and sickness absence. The aim of this review was to explore and uncover this relationship by looking at differences in type of design (cross-sectional vs. longitudinal), type of data (self-reported vs. registered data), and type of sickness absence (long-term vs. short term).

**Method:**

Six databases were searched through June 2020. Observational and experimental studies from 1980 to 2020, in English or Scandinavian languages reporting the results of the association between alcohol consumption and sickness absence among working population were included. Quality assessment, and statistical analysis focusing on differences in the likelihood of sickness absence on subgroup levels were performed on each association, not on each study. Differences in the likelihood of sickness absence were analyzed by means of meta-analysis. PROSPERO registration number: CRD42018112078.

**Results:**

Fifty-nine studies (58% longitudinal) including 439,209 employees (min. 43, max. 77,746) from 15 countries were included. Most associations indicating positive and statistically significant results were based on longitudinal data (70%) and confirmed the strong/causal relationship between alcohol use and sickness absence. The meta-analysis included eight studies (ten samples). The increased risk for sickness absence was likely to be found in cross-sectional studies (OR: 8.28, 95% CI: 6.33–10.81), studies using self-reported absence data (OR: 5.16, 95% CI: 3.16–8.45), and those reporting short-term sickness absence (OR: 4.84, 95% CI: 2.73–8.60).

**Conclusion:**

This review supports, but also challenges earlier evidence on the association between alcohol use and sickness absence. Certain types of design, data, and types of sickness absence may produce large effects. Hence, to investigate the actual association between alcohol and sickness absence, research should produce and review longitudinal designed studies using registry data and do subgroup analyses that cover and explain variability of this association.

## Introduction

Alcohol is the most used and misused psychoactive substance in the general population as well as in the workforce [1]. Studies have indicated that one to three out of ten employees may benefit from alcohol prevention interventions due to risky drinking [2, 3] (i.e., a drinking pattern that increases the likelihood of social, medical, occupational, and economic problems [4]). For decades, alcohol-related problems and risky drinking among employees has been attracting interest, as well as raising concerns among researchers, organizations, and practitioners [5, 6]. Concerns are mainly due to the increased prevalence of on-the-job impairment (i.e., working under the influence of alcohol (on-the-job drinking)), and impact of risky drinking during nonworking hours (off-the-job drinking) on work performance [7].

Evidence has demonstrated that drinking alcohol may facilitate social interactions [8, 9] or can cover up negative emotions [10]. However, alcohol consumption among employees (on-the-job / off-the-job drinking) has been associated with a variety of detrimental outcomes, with regards to productivity (e.g., impaired work performance in terms of presenteeism [11, 12]), work environment (e.g., social exclusion, unwanted sexual attention, and verbal abuse [13]), and behavioral changes [14], depending on the level of drinking. Defined standard alcohol units and thresholds for at-risk drinking vary considerably across countries, regions, industries, and work groups, depending on the nature of work, existing regional culture, ease of access to alcohol, and work environment [15–17]. There is inconsistent evidence with respect to the relationship between different drinking patterns and adverse outcomes [18, 19]. Hence, a more detailed knowledge about the specific characteristics and context of different drinking patterns may be helpful in our understanding of the consequences of risky drinking [20].

Sickness absence is a major public health concern in many countries since it leads to problems not only for the individual in question, but also for the workplace, family life and the surrounding peer groups and society [21]. Furthermore, it can impose a substantial financial burden on both the individual and the community (i.e., workplace and society) [22]. For example, the cost of sickness absence is estimated at $2,660 per year for salaried employees in the USA, and about 2.5% of GDP in Europe [23, 24]. Sickness absence is a significant issue influenced by various factors, comprising personal (e.g., individual’s health behaviors, socioeconomic status, or evaluation of own health), and contextual factors (e.g., existing health care system, absence policies and benefits, work conditions, and supervisor support) [25–27]. These factors may influence type and duration of one’s reported sickness absence. For example, existing sickness absence benefit systems in each country may affect the evaluation of one’s own health in regards to when and how long sickness absence is needed. This, in turn, may affect the reported sickness absence as being registered/certified (mostly long-term sickness absence) or becoming a self-reported one (mostly short-term sickness absence) [27, 28]. Dale-Olsen and Markussen [29] focused on the trends in absenteeism for a period from 1972 to 2008 in Norway, which is known for having a generous sickness absence benefit system [27]. Authors found that although the duration of each spell was increased by 20% for specific diagnoses, the number of sick leave spells was not changed.

Several studies have explored the relationship between different measures of alcohol consumption and sickness absence in working populations. Alcohol-related sickness absence often includes being late for work, being on partial absence during the workday, leaving early, one-day absences due to hangover, or being absent for several days [30]. Studies from Norway reported that between 14% and 50% of the total short-term absence days (1–3 days) could be linked to alcohol [31, 32]. Cunradi et al. [33] found short-term sickness absence to be associated with problem drinking. Roche et al. [34] found an association between risky drinking (compared to low-risk drinking) and self-reported sickness absence. Although self-reported sickness absence becomes less reliable when days of absence increase, but its sensitivity is acceptable as long as the length of absences not exceeding one week [35]. Moreover, although a significant association between registered absence and various measures of health has been shown [36–38], access to registered data can be problematic, and that makes many studies rely on self-reported sickness absence data.

Systematic reviews and meta-analyses have found fairly strong evidence for the association between alcohol consumption and sickness absence [39–41]. However, these studies were based on observational data and did not differentiate between heterogenous measures of alcohol consumption and sickness absence that vary in content and comparability. Based on earlier research, it is evident that there is a measurement challenge in sickness absence and presenteeism research, with high variability of measurement approaches concerning sickness absence levels (e.g., collapsing all types of sickness absence together) [11, 39] and differences in sickness absence benefit systems [27, 42]. Therefore, these concerns make the reported relationships between alcohol consumption and sickness absence in the literature “a black box” that needs to be investigated, by looking into subgroups including measurement groupings and type of data. Hence, the aim of this systematic review and meta-analysis was to explore and uncover the relationship between alcohol use patterns and sickness absence by looking at differences in type of design (cross-sectional vs. longitudinal), type of data (self-reported vs. registered data), and type of sickness absence (long-term vs. short term).

## Methods

### Protocol and registration

This study was designed as a systematic review and meta-analysis based on the Cochrane recommendations [43]. The review protocol was registered in the International Prospective Register of Systematic Reviews (PROSPERO; registration number: CRD42018112078, registration date: 29/10/18) [44]. This paper is reported in accordance with the PRISMA (Preferred Reporting Items for Systematic Reviews and Meta-Analyses) guidelines (S1 File) [45].

### Eligibility criteria

Studies exploring the relationship between alcohol consumption and sickness absence among employees were included. Studies had to satisfy the following criteria: (i) *study design* (quantitative studies; observational and experimental designs), (ii) *type of participants* (all salaried persons, hired and self-employed), (iii) *type of measures/tests* (reporting results from one or more statistical tests of an association between alcohol consumption and sickness absence, (iv) *type of publication* (full-text research article published in scientific peer reviewed journal), (v) *language* (published in English or a Scandinavian (Norwegian, Swedish or Danish) language, and (vi) *time* (published year 1980 or later).

In order to be included in the meta-analysis, studies additionally had to satisfy the following criteria: (vii) reporting data on event/participants that could be converted to odds ratios (ORs) (i.e., reporting the number of alcohol drinking participants having sickness absence), and (viii) reporting results for at least two categories of alcohol intake levels (including a category of non-alcohol intake/occasional/low alcohol intake as a reference category, a category of moderate drinking, or a category of risky/problem/heavy drinking).

### Databases and search strategy

A search strategy was developed and utilized in six scientific databases (Medline, Embase, Cinahl, PsycInfo, AMED, and Web of Science). Where appropriate, the strategy was adapted to each database to ensure comparability. The search strategy consisted of abstract-level text searches and MeSH terms (Medical Subject Headings, Topics, or similar terms), and comprised two thematic blocks: (i) alcohol consumption (drink* OR alcohol* OR drunk* OR hangover OR “hang over” OR alcohol drinking (MeSH) OR binge drinking (MeSH)), and (ii) sickness absence (“sick leave” OR “sickness absence” OR absenteeism OR “lost work days” OR “lost work hours” OR “leave of absence” OR “work absence” OR “illness days” OR absenteeism (MeSH) OR sickness absence (MeSH) OR sick leave (MeSH)) (S1 Table). The two search blocks were then combined (using the Boolean operator AND), and search results were transferred to EndNote.

Databases were searched through June 2020. Additionally, manual searches for potentially relevant studies were performed in Google Scholar and Research Gate, by two reviewers (NSH and MMT) in reference lists for the included studies (ancestry approach).

### Study selection

Identified searches were screened for relevance on a title/abstract level, and potentially relevant studies were assessed in full-text format independently by two reviewers (NSH and AS). A third reviewer (RWA) served as a tiebreaker in case of disagreement. Next, two reviewers independently assessed all eligible studies for inclusion in the meta-analysis (NSH and JCS). Reviewers contacted studies’ authors reporting odds ratios or risk ratios to get detailed data (according to criteria vii). Although a few authors responded, none of them had access to the asked information.

### Data extraction

Relevant information was extracted independently by two reviewers for all studies (NSH and AS) and those deemed eligible for inclusion in the meta-analysis (NSH and JCS). Among studies reporting different types of sickness absence, results for alcohol use and sickness absence were extracted, but other types e.g., specific subgroups of injury/illness-related sickness absence (e.g., accident or mental disorder) were discarded. As the included studies used somewhat dissimilar alcohol consumption measures, standardization was necessary. Therefore, alcohol consumption was converted into grams of ethanol per day by means of the following formula: 1 ml = 0.8 grams, and 1 standard drink (SD) = 10.0 grams/day [46]. Hence, the measure of alcohol consumption was defined using the following: light consumption (< 1 drink/day), moderate consumption (< 2 drinks/day), and risky consumption (≥ 2 drinks/day) [47, 48]. Abstainers were excluded as this information was not reported in all studies. Furthermore, as moderate drinking was not measured in all studies, alcohol consumption was categorized into two groups: low-risk (reference group; comprised light-to-moderate drinking) and risky drinking. Studies not reporting grams of alcohol (e.g., reporting units), were converted to grams according to each study’s national guidelines [16].

### Quality assessment

Quality of the included data were assessed independently by two reviewers (NSH and MMT). Quality assessments were performed on associations rather than on studies, as the included studies often tested more than one statistical association between alcohol consumption and sickness absence. This approach is in line with the procedures applied in earlier systematic reviews of relationships between alcohol consumption and occupational outcomes among employees [11, 39].

A modified version of the Newcastle-Ottawa Scales (NOS) was utilized [49, 50], and associations were assessed on five key domains: (i) representativeness of the sample (low quality = non-random sample or inadequate description; high quality = probability or non-probability sampling procedure), (ii) measure of alcohol consumption (low quality = non-validated self-reported measure or inadequate description; high quality = validated self-report instrument (e.g., AUDIT) or objective measure (e.g., CDT blood test)), (iii) measure of sickness absence (low quality = self-reported or inadequate description; high quality = record linkage (register data)), (iv) level of adjustment (low quality = unadjusted or unclear; high quality = adjusted for at least one individual (e.g., sociodemographic) and/or one environmental (e.g., work-related) factor), and (v) test description (low quality = inadequate description or missing key information (e.g., likelihood, *p*-value); high quality = adequate description of key information). The quality assessment procedure was piloted on a random sample of 10 associations and evaluated prior to quality assessment of all included data.

### Analysis

An overall assessment on the association between alcohol consumption and sickness absence was conducted by looking into descriptive characteristics of the included studies. Tested associations between alcohol consumption and sickness absence reported by the included studies were analyzed descriptively in different subgroups based on:

- Type of design,
- Direction of associations (statistically significant positive; neutral (i.e., no association); statistically significant negative), which further were categorized based on direction (positive; negative) and statistical significance (significant; non-significant),
- Type of measurement/operationalization (alcohol: frequency and quantity, volume per day, average drinking per week, heavy episodic/binge drinking (i.e., six or more drinks on one occasion [4]), diagnosed problem drinking, and sales of pure alcohol; sickness absence: total number of absence days (i.e., total number of days of sickness absence per year), short-term absence (varied in studies from ≤ 3 days to < 7 days), and long-term absence (varied in studies from ≥ 3 days to ≥ 7 days)).

Eight studies including ten samples satisfying the additional inclusion criteria (criteria vii and viii above) were subjected to meta-analysis in the RevMan 5 software [43]. Due to heterogeneity between studies, a random-effects model was applied to calculate summarized odds ratios (OR) with 95% confidence intervals (CI) as an overall synthesized measure of pooled estimate [51]. All reported raw data, e.g., number of participants at risk (for each level of alcohol use) and number of events (participants at risk reporting sickness absence) were collected from the ten samples in the meta-analyses. Then it was possible to calculate effect measures as odds ratio or relative risk (RR), avoiding re-calculation between different effect measurements. The Cochrane handbook suggests using either OR or RR. Therefore, OR was chosen to be used rather than RR due to being often used in this field. The DerSimonian-Laird estimator implemented in the RevMan 5 software was used to calculate the between-study variance. Forest plots were created for risky drinking versus low-risk drinking. The L’Abbe plot [52] was used to compare studies’ likelihood rates (log ORs) among low-risk and risky drinking employees. Heterogeneity across studies was explored using a chi-square statistic (χ^2^) and *I*^*2*^-test. Considerable heterogeneity was deemed present at *I*^*2*^ > 50% [53].

The main results were extracted from the statistical subgroup analyses. Subgroup analyses were applied to identify sources of heterogeneity, as well as to explore the differences on the association between alcohol and sickness absence across different categories. These analyses were performed according to studies and participants’ characteristics including type of study design, sickness absence measure, sickness absence duration, year of publication, and country. Sensitivity analyses were performed on both the descriptive part and meta-analysis part. For the meta-analysis part, sensitivity analyses were performed by omitting one study and calculating the pooled ORs for the remaining studies. Publication bias was examined running a funnel plot and by using a Harbord regression-based test to explore funnel plot asymmetry [54].

In studies reporting outcomes from independent groups (e.g., short- or long-term absences), each group was added as a separate sample in the meta-analysis. Additional tests (Harbord regression-based test) and the L’Abbe plot were performed with Stata version 16.0 [55].

## Results

### Overview of the evidence

**Fig 1:**
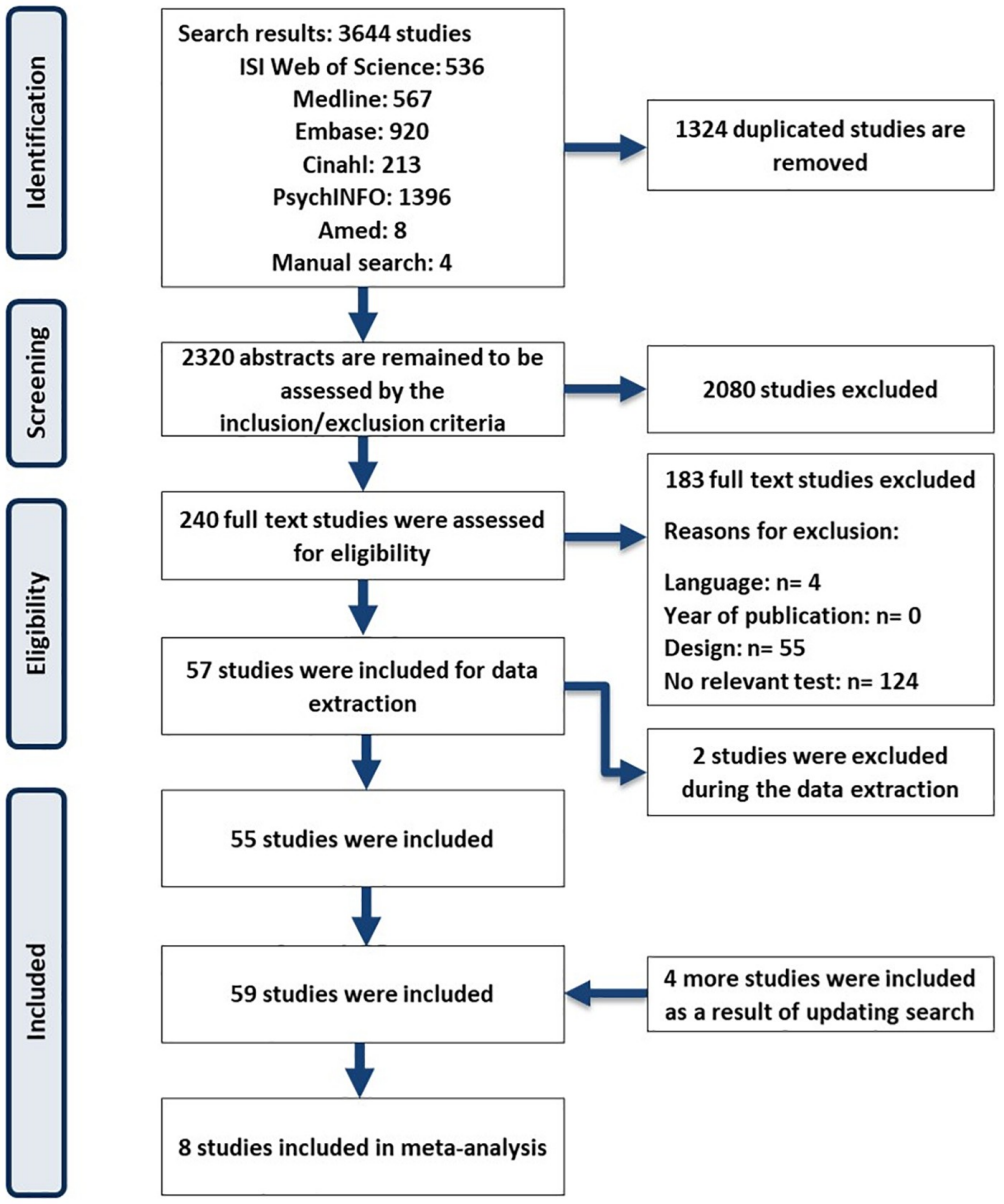
Flowchart for the search and study selection process.

A total of 3,644 studies were identified (Fig 1). After duplicate removal (*n* = 1,324) and excluding 2,080 studies that did not fulfill the inclusion criterion (e.g., no relevant test or study design), 240 articles were assessed for eligibility in full-text format, resulting in 55 included studies. Four more studies were included as a result of updated searches in June 2020. Finally, 59 studies were included in the systematic review. Eight studies met the inclusion criteria for meta-analysis [21, 33, 34, 56–62].

An overview of the eligible studies including the sample settings, study designs, measures of the predictor and outcome, along with the number of tested associations on alcohol consumption and sickness absence in each study can be seen in Table 1. Tested associations can be found in S2 Table using association IDs. Almost 91.5% of studies (54 out of 59) were observational studies (cross-sectional: *n* = 17; longitudinal: *n* = 37, including 29 cohort studies, 7 panel studies, and 1 case-control study) and the remaining five were based on experimental designs (randomized controlled studies: *n* = 1, and quasi-experimental (time-series) studies: *n* = 4) (Table 1). The 59 studies comprised a total sample size of 439,209 employees (ranging between 43 and 77,746). Studies originated from 15 different countries: Sweden (*n* = 12), Finland (*n* = 12), USA (*n* = 9), Norway (*n* = 7), Australia (*n* = 3), Denmark (*n* = 3), United Kingdom (*n* = 3), Brazil (*n* = 2), Belgium (*n* = 1), Japan (*n* = 2), Ethiopia (*n* = 1), France (*n* = 1), India (*n* = 1), Netherlands (*n* = 1), and Uganda (*n* = 1). Type of working environments varied in included studies. Working environments consisted of participants employed in e.g., police stations [63, 64], transport services [56, 65], hospitals [66], farm industries [67], etc. A total of 162 associations between measures of alcohol consumption and sickness absence were tested in these 59 included studies.

**Table 1:** Overview of included studies (n = 59), associations (n = 162), and measurements.

Study (author, year)	Sample	Design	Alcohol measure	Sickness absence measure	Tested associations, *n* (association ID)
Jenkins (1986) [68]	UK: civil servants (*n* = 321)	Longitudinal (cohort)	Drinking during the last 7 days (frequency and quantity)	Company-registered certified and uncertified absence days	1 (1)
Persson & Magnusson (1989) [69]	Sweden: adult patients (*n* = 2,038)	Longitudinal (panel)	Excessive drinking (>280 g ethanol per week) / high alcohol level in blood / doctor diagnosis	National-registered sickness absence days during the 5 different years	2 (2, 3)
Marmot et al. (1993) [70]	UK: non-industrial civil servants (*n* = 10,314)	Longitudinal (cohort)	Frequency of drinking during the last year and last 7 days	Self-reported and registered short spells (<7 days) and long spells (>7days)	4 (4–7)
North et al. (1993) [71]	UK: non-industrial civil servants (*n* = 10,314)	Longitudinal (cohort)	Frequency of drinking during the last year and last 7 days	Self-reported and registered short spells (<7 days) and long spells (>7days)	4 (8–11)
Blum (1993) [72]	USA: employees (*n* = 136)	Cross-sectional	Drinking during the last 7 days (frequency and quantity)	Self-reported days of absence (last 2 weeks)	3 (12–14)
French et al. (1995) [73]	USA: employees in five different worksites (*n* = 1,664)	Cross-sectional	Number of drinks during the last year	Self-reported absence days during the last year	1 (15)
Vasse et al. (1998) [74]	Netherlands: employees in various occupations (*n* = 471)	Cross-sectional	Drinking during the last 6 months (frequency and quantity)	Self-reported sickness absence spells during the last 6 months (yes/no)	2 (16, 17)
Spak et al. (1998) [75]	Sweden: general population (*n* = 3,130)	Cross-sectional	Diagnosed problem drinking	National-registered days of absence during the last year	3 (18–20)
Upmark et al. (1999) [76]	Sweden: general population (*n* = 1,855)	Longitudinal (cohort)	Average of drinking during the last week/ problem drinking (CAGE score)	National-registered days of absence per year	8 (21–28)
Upmark et al. (1999) [77]	Sweden: mandatory conscripts (*n* = 8,122)	Longitudinal (cohort)	Problem drinking (>250 g ethanol per week)/ periods of frequent drunkenness	National-registered number of absence days	3 (29–31)
Richmond et al. (1999) [63]	Australia: police employees (*n* = 954)	Experimental (RCT)	Average weekly consumption (frequency and quantity) / binge drinking	Self-reported number of absence days	2 (32, 33)
Holder and Blose (1991) [78]	USA: manufacture employees (*n* = 3,656)	Longitudinal (cohort)	Diagnosed problem drinking	Registered number of absence days during the last year	1 (34)
Vahtera et al. (2002) [37]	Finland: municipal employees (*n* = 6,442)	Longitudinal (cohort)	Drinking (frequency and quantity)	Company-registered medically certified sickness absence days	1 (35)
Hermansson et al. (2002) [56]	Sweden: transport employees (*n* = 989)	Longitudinal (cohort)	Problem drinking: AUDIT^a^ / CDT^b^ (blood test) / GGT^c^	Company-registered sickness absence days	3 (36–38)
McFarlin & Fals-Stewart (2002) [79]	USA: employees in various occupations (*n* = 280)	Cross-sectional	Drinking days during the last month	Company-registered sickness absence days	3 (39–41)
Kivimäki et al. (2002) [36]	Finland: municipal employees (*n* = 2,991)	Longitudinal (panel)	Drinking (frequency and quantity) / alcohol intoxication	Company-registered sickness absence days	4 (42–45)
Bendtsen et al. (2003) [80]	Sweden: employees in various occupations (*n* = 1,075)	Cross-sectional	Frequency of alcohol intake/ increased consumption last year	Registered sickness absence days and spells	3 (46–48)
Morikawa et al. (2004) [81]	Japan and UK: employees (*n* = 8,794)	Longitudinal (cohort)	Average drinks per week	Registered long-term sickness absence days (>7 days)	4 (49–52)
Voss et al. (2004) [82]	Sweden: post employees (*n* = 3,470)	Cross-sectional	Alcohol consumption	Company-registered sickness absence days	2 (53, 54)
Cunradi et al. (2005) [33]	USA: municipal transit operators (*n* = 1,446)	Longitudinal (cohort)	Average alcohol intake / problem drinking CAGE	Self-reported short-term sickness absence	4 (55–58)
Floderus et al. (2005) [83]	Sweden: employees (*n* = 862)	Cross-sectional	Alcohol consumption	National-registered long-term sickness absence	1 (59)
Ovuga & Madrama (2006) [64]	Uganda: police officers (*n* = 104)	Cross-sectional	prevalence of probableAUD^d^ and prevalence of alcohol use problems (AUP)	Self-reported sickness absence during the past 3 months	2 (60, 61)
Pidd et al. (2006) [84]	Australia: employees in various occupations (*n* = 11,608)	Cross-sectional	Frequency and amount of drinking	Self-reported sickness absence days	2 (62, 63)
Kondo et al. (2006) [57]	Japan: electronic employees (*n* = 1,183)	Longitudinal (panel)	Number of drinks per week	Self-reported sickness absence of 5 days or longer	2 (64, 65)
Kujala et al. (2006) [21]	Finland: employees (*n* = 3,725)	Longitudinal (cohort)	Amount of consumed alcohol per day (volume)	National-registered medically certified long-term sickness absence (>9 days)	2 (66, 67)
Norström (2006) [85]	Sweden: employees (*n* = not vailable)	Experimental (Quasi)	Alcohol consumption was gathered by sales of pure alcohol (100%) per capita	Self-reported and national registered sickness absence days	2 (68, 69)
Christensen et al. (2007) [86]	Denmark: employees (*n* = 5,020)	Longitudinal (cohort)	Alcohol consumption	National- registered long-term (>7 weeks) sickness absence	2 (70, 71)
Suominen et al. (2007) [87]	Finland: non-industrialized employees (*n* = 5,000)	Longitudinal (cohort)	Frequency of high alcohol consumption	National-registered sickness absence spells (> 8 days)	1 (72)
Johansson et al. (2009) [88]	Finland: general population (*n* = 5,000)	Longitudinal (panel)	Average of consumed units per week	Self-reported sickness absence during the last year	1 (73)
Laaksonen et al. (2009) [58]	Finland: municipal employees (*n* = 6,934)	Cross-sectional	Average of consumed units per week	Self-reported and registered sickness absence spells	4 (74–77)
Roche et al. (2008) [34]	Australia: employees (*n* = 13,582)	Cross-sectional	Frequency and amount of drinking during the last week	Self-reported and registered sickness absence (last 3 months)	2 (78, 79)
Salonsalmi et al. (2009) [89]	Finland: municipal employees (*n* = 6,509)	Longitudinal (cohort)	Average units per week / binge drinking / CAGE	Self-reported and national-registered sickness absence spells	12 (80–91)
Norström & Moan (2009) [90]	Norway: manual workers (*n* = not available)	Experimental (Quasi)	Alcohol consumption was gathered by sales of pure alcohol (100%) per capita	National-registered percentage of sickness absence days	2 (92, 93)
Bacharach et al. (2010) [65]	USA: transport employees (*n* = 470)	Longitudinal (cohort)	Frequency and average amount of drinking / binge drinking	Company-registered sickness absence days	2 (94, 95)
Balsa & French (2010) [91]	USA: general population (*n* = 6,015)	Experimental (Quasi)	Heavy drinking: intoxicating / alcohol dependence DSM-IV	Self-reported number of sickness absence days	3 (96–98)
Kirkham et al. (2015) [92]	USA: computer manufacturer employees (*n* = 17,089)	Longitudinal (cohort)	Problem drinking (CAGE)	Company-registered sickness absence days	1 (99)
Hensing et al. (2011) [59]	Sweden: sick listed and general population (*n* = 6,455)	Cross-sectional	Drinking during the last 12 months, problem drinking (AUDIT)	Self-reported absence spells	2 (100, 101)
Edvardsen et al. (2015) [93]	Norway: employees in various occupations (*n* = 2,437)	Cross-sectional	Self-reported consumption last 24 hours / oral fluid samples	Self-reported absence days	4 (102–105)
Lidwall & Marklund (2011) [94]	Sweden: employees in various occupations (*n* = not available)	Longitudinal (panel)	Amount of alcohol consumption	Self-reported and registered long-term sickness absence	2 (106, 107)
Chakraborty & Subramanya (2013) [66]	India: hospital employees in psychiatric department (*n* = 43)	Cross-sectional	Alcohol abuse/ dependence	Self-reported sickness absence days	1 (108)
Schou et al. (2014) [95]	Norway: young employees (*n* = 1,762)	Longitudinal (cohort)	Frequency of drinking / intoxication last year	Self-reported sickness absence (yes/no)	2 (109, 110)
Ervasti et al. (2018) [96]	Finland, France, UK: employees in various occupations (*n* = 46,514)	Longitudinal (cohort)	Weekly alcohol consumption	Registered days of sickness absence per year	1 (111)
Ervasti et al. (2018) [97]	Finland, France, UK: employees in various occupations (*n* = 47,520)	Longitudinal (cohort)	Weekly alcohol consumption	Registered sickness absence days	1 (112)
Torvik et al. (2016) [98]	Norway: young employees (*n* = 2,178)	Longitudinal (cohort)	Alcohol use disorder (DSM-IV)	National-registered sickness absence days	1 (113)
Silva-Junior & Fischer (2014) [99]	Brazil: public social security branch (*n* = 385)	Longitudinal (case-control)	Problem drinking (AUDIT)	National-registered long-term sickness absence	1 (114)
Richmond et al. (2016) [100]	USA: employees in various occupations (*n* = 338)	Experimental (Quasi)	Problem drinking (AUDIT)	Self-reported sickness absence days	1 (115)
De Clercq et al. (2015) [101]	Belgium: employees (*n* = 24,402)	Longitudinal (cohort)	Alcohol consumption (more than 3 units of alcohol per day)	Company-registered absence at least 10 days in a 12-month period	1 (116)
Østby et al. (2016) [102]	Norway: young adult twins (*n* = 6,735)	Longitudinal (panel)	Frequency of alcohol use during the last 14 days / binge drinking	Registered sickness absence days	2 (117, 118)
Morois et al. (2017) [103]	France: French national electricity and gas company (*n* = 9,907)	Longitudinal (cohort)	Daily alcohol consumption (gram/day)	Company-registered short-term (<8 days), moderate (8–28 days), and long-term (>28days)	6 (119–124)
Ervasti et al. (2018) [104]	Finland: public sector employees (*n* = 5,809)	Longitudinal (cohort)	Weekly alcohol use	Registered short-term absence	4 (125–128)
Salonsalmi et al. (2015) [105]	Finland: middle-aged employees (*n* = 8,960)	Longitudinal (panel)	Weekly average consumption/ problem drinking (CAGE)	Self-reported and company registered sickness absence spells, self-certified and medically confirmed (4+ days)	8 (129–136)
Araujo et al. (2017) [106]	Brazil: employees (*n* = 342)	Longitudinal (cohort)	Weekly frequency of drinking	Self-reported sickness absence days	1 (137)
Schou & Birkelund (2015) [107]	Norway: young employees (*n* = 1,460)	Longitudinal (cohort)	Frequency of alcohol consumption / heavy drinking / intoxicating	National-registered sickness absence days	6 (138–143)
Kaila Kangas et al. (2018) [60]	Finland: general population (*n* = 3,666)	Longitudinal (cohort)	Amount of drinking/ alcohol use disorder	National-registered sickness absence days	2 (144, 145)
Jørgensen et al. (2017) [61]	Denmark: general adult population (*n* = 17,690)	Longitudinal (cohort)	Frequency and amount of drinking during the last week / binge drinking	National-registered sickness absence days	4 (146–149)
Jørgensen et al. (2019) [62]	Denmark: general adult population (*n* = 77,746)	Longitudinal (cohort)	Frequency and amount of drinking during the last week, problem drinking (CAGE-C)	National-registered sickness absence days	2 (150, 151)
Lund et al. (2019) [108]	Norway: employees (*n* = 1,870)	Cross-sectional	Binge drinking	Self-reported sickness absence days in the last 12 months	2 (152, 153)
Hambisa Mekonnen et al. (2019) [67]	Ethiopia: farm industry workers (*n* = 444)	Cross-sectional	Frequency and amount of drinking	Company registered sickness absence days	1 (154)
Landberg et al. (2020) [109]	Sweden: adult employees (*n* = 15,983)	Longitudinal (cohort)	Average weekly volume and frequency of heavy episodic drinking	Self-reported short-term and national-registered long-term (>14 days) sickness absence	8 (155–162)

^a^ AUDIT: Alcohol Use Disorder Identification Test;

^b^ CDT: Carbohydrate-Deficient Transferrin test;

^c^ GGT: Gamma-glutamyl Transferase test;

^d^ AUD: Alcohol Use Disorder.

### Associations between alcohol consumption and sickness absence

Out of 162 tested associations, 148 (91%) indicated that higher levels of alcohol consumption were associated with higher levels of sickness absence (positive associations), while 14 (9%) indicated a negative relationship, i.e., that higher levels of alcohol consumption were associated with lower levels of sickness absence (Table 2 and S2 Table). About 63.5% (*n* = 94) of positive associations and none of negative associations were statistically significant. The majority of associations with positive and statistically significant results were based on longitudinal data (66 of 94, 70%).

**Table 2:** Tested associations (n = 162) according to measurements of alcohol consumption and sickness absence.

Alcohol measure		Sickness absence measure					
		Total number of absence days		Short-term absence		Long-term absence	
		Pos.	Neg.	Pos.	Neg.	Pos.	Neg.
Frequency and quantity	sig.	[1], [13], [14], [15], [29], [40], [53], [78], [79], [102], [104], [105], [109], [111], [138], [140], [147], [150], and [162]	None	[8], [9], [62], [125], [127], and [128]	None	[10], [35], [47], [48], [63], [106], [107], [112], [116], and [117]	None
	ns.	[12], [17], [39], [41], [42], [54], [94], [137], [142], and [146]	[16], [43], and [103]	[4] and [126]	[5]	[6], [11], [70], [71], [106], and [144]	[7], [59], [64], [65], [72], and [100]
Volume per day	sig.	[119] and [120]	None	[121] and [122]	None	[123] and [124]	None
	ns.	None	[67]	None	None	None	[66]
Average drinking per week	sig.	[21], [22], [32], and [73]	None	[57], [74], [75], [80], [129], [154], and [155]	None	[50], [52], [86], [158], and [159]	None
	ns.	[23] and [24]	None	[56], [81], and [130]	None	[49], [51], [76], [77], [87], [133], and [134]	None
Heavy episodic / binge drinking	sig.	[33] and [95]	None	[82], [83], [156], and [157]	None	[88], [118], and [160]	None
	ns.	[148] and [149]	None	[152]	None	[89], [153], and [161]	None
Diagnosed problem drinking	sig.	[2], [3], [18], [19], [20], [30], [34], [44], [61], [98], [108], [110], [115], [139], [143], and [151]	None	[55], [58], [84], [85], and [131]	None	[36], [90], [91], [101], [114], and [145]	None
	ns.	[26], [27], [28], [31], [45], [60], [97], [99], and [141]	[25] and [96]	[132]	None	[37], [38], [113], [135], and [136]	None
Drinking based on sales of pure alcohol	sig.	[68] and [92]	None	None	None	None	None
	ns.	[69] and [93]	None	None	None	None	None

[numbers] = association IDs; Pos. = positive direction; Neg. = negative direction; ns = non-significant association; sig. = significant association; For instance: association [1] (upper left in the table) was a statistically significant positive association between sickness absence (measured in terms of total number of absence days) and alcohol consumption (measured in terms of frequency and quantity).

Regarding the type of alcohol measures, frequency, and quantity (39%) as well as problem drinking (27%) were the most frequently applied. More than half of the associations between frequency and quantity of alcohol consumption and sickness absence (36 of 63) were statistically significant (Table 2). Six out of eight (75%) associations on volume of drinking per day and likelihood of sickness absence revealed significant results. Nine of 15 associations (60%) exploring binge drinking and sickness absence reported significant associations. In terms of type of sickness absence measures, almost half of the associations (76 out of 162) used total number of absence days to measure sickness absence. Roughly 33% (*n* = 54) of associations used long-term and the remaining 20% (*n* = 32) used short-term absences. More than half of associations (44 of 76) between alcohol measures and total number of reported absence days were significant. Three-quarters of the associations (24 of 32) on alcohol and short-term absences and almost half of associations (26 of 54) on alcohol and long-term absences were significant.

### Likelihood of sickness absence among risky drinking employees versus those with low-risk drinking

**Fig 2:**
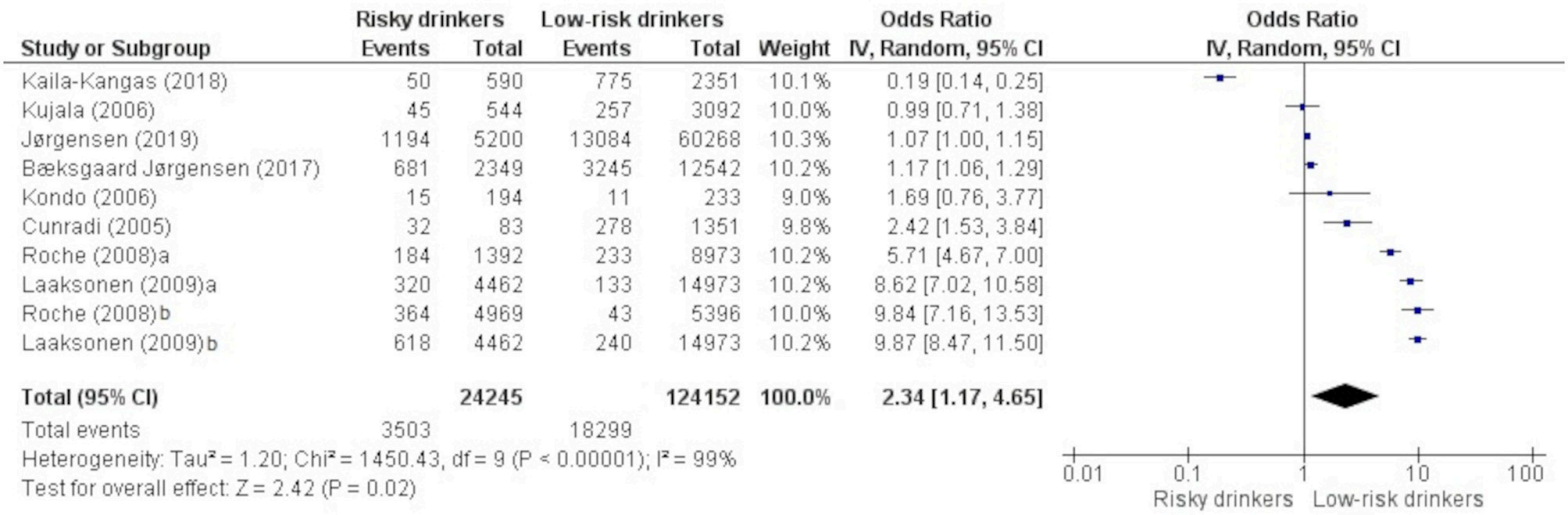
Pooled odds estimate for sickness absence among risky drinking employees versus those with low-risk drinking.

Altogether, 10 samples (from eight studies) were included in the meta-analysis. A synthesis of samples showed that risky drinking was associated with an increased odd of sickness absence (OR: 2.34, 95% CI: 1.17–4.65), see Fig 2. Very high levels of heterogeneity existed between studies included in the overall estimate (χ^2^ = 1450.43, *P*< .00001, *I*^*2*^ = 99%).

**Fig 3:**
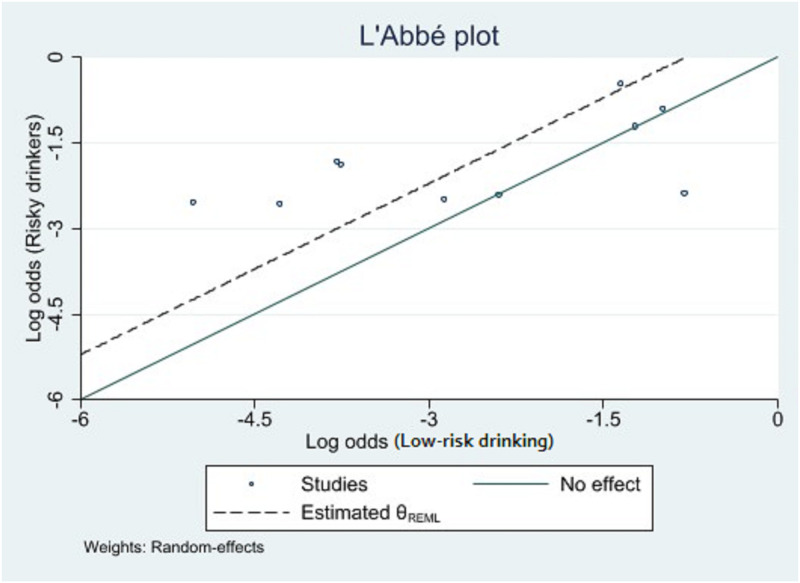
L’Abbé plot of comparing likelihood rates in low-risk and risky drinking employees.

As shown in the L’Abbé plot (Fig 3), seven samples were above the no effect line, suggesting that the likelihood of sickness absence was higher among risky drinking employees than those with low-risk drinking, compared to the sample below the line.

#### Subgroup analyses

Subgroup analyses indicated that sickness absence was more likely among the risky drinking employees than low-risk ones in studies employing cross-sectional designs (OR: 8.28, 95% CI: 6.33–10.81), self-reported absence data (OR: 5.16, 95% CI: 3.16–8.45), short-term absence data (OR: 4.84, 95% CI: 2.73–8.60), as well as studies conducted in the USA (OR: 2.42, 95% CI: 1.53–3.84) and Australia (OR: 7.41, 95% CI: 4.15–13.21) (Table 3 and S1–S5 Figs).

**Table 3:** Pooled odds ratio (OR) and 95% CI for alcohol intake and likelihood of sickness absence, stratified by selected covariates.

Factors	Number of studies	OR (95% CI)	*I*^*2*^ (%)	*P*-value ^a^
**All studies**	10	2.34 (1.17–4.65)	99.0	*P* < .00001
**Study design**				
Cross-sectional	4	8.28 (6.33–10.81)	98.8	*P* < .00001
Longitudinal	6	0.94 (0.64–1.39)		
**Sickness absence measurement**				
Self-reported	5	5.16 (3.16–8.45)	91.3	*P* < .0001
Registered	5	1.16 (0.57–2.36)		
**Sickness absence duration**				
Long-term	4	1.80 (0.32–10.32)	92.0	*P* < .00001
Short-term	4	4.84 (2.73–8.60)		
Number of days	2	1.11 (1.03–1.21)		
**Year of publication**				
2000–2008	5	3.02 (1.28–7.12)	0.0	*P* = .45
2009–2019	5	1.83 (0.70–4.83)		
**Region**				
USA	1	2.42 (1.53–3.84)	92.2	*P* < .00001
Japan	1	1.69 (0.76–3.77)		
Australia	2	7.41 (4.15–13.21)		
Finland	4	2.01 (0.35–11.56)		
Denmark	2	1.11 (1.03–1.21)		

^a^ Test for subgroup differences.

#### Sensitivity analyses

Omitting each study in turn did not change the tendency of the ORs. However, after omitting one (Roche (2008b) of the 10 samples from the meta-analysis, the pooled estimate was rendered non-significant (OR: 1.99, 95% CI: 0.98–4.05). This sample was based on the association between consumption during single drinking occasions (episodic drinking) and sickness absence. This sample had an approximately equal proportion of risky drinkers and low-risk drinkers (Fig 2), while in the other samples the higher proportion were low-risk drinkers. Moreover, one study was based on all-cause sickness absence (e.g., certified sickness absence due to mental- or musculoskeletal disorder) [60]. Conducted sensitivity analysis found stronger alcohol-absence association after omitting this study (OR: 3.10, 95% CI: 1.56–6.17).

In addition, five out of 59 included studies measured sickness absence using self-reported alcohol-related sickness absence [34, 73, 84, 95, 107]. After omitting these studies, still the majority of the tested associations (140 of 162) indicated that higher levels of alcohol consumption were associated with higher levels of sickness absence and about 61.4% of them (86 of 140) were statistically significant.

#### Publication bias

**Fig 4:**
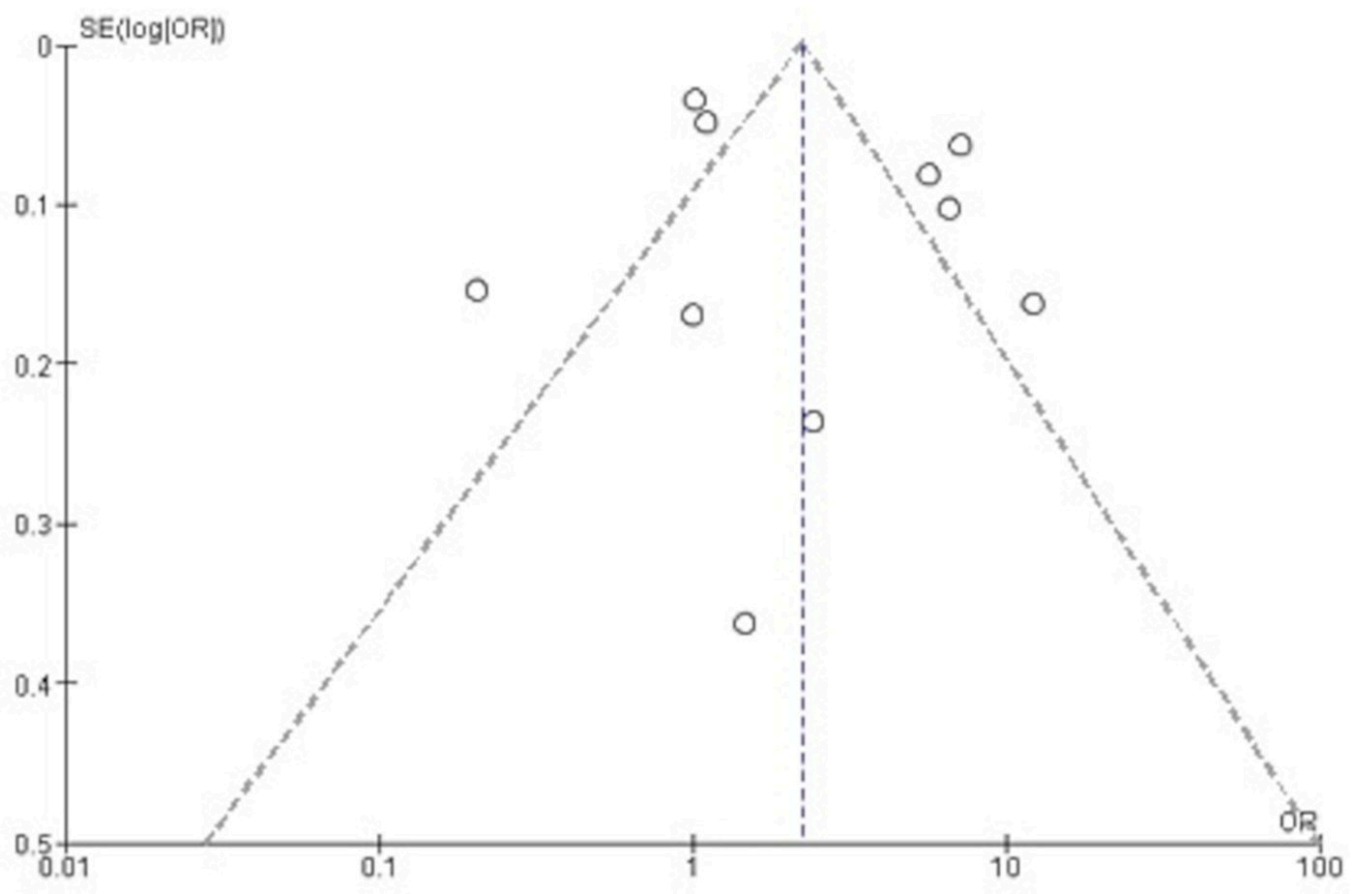
Funnel plot of publication bias.

Visual inspection of the funnel plot indicated a symmetric shape around the weighted average effect size, yielding little support for publication bias, see Fig 4. Only two samples resided within the pseudo 95% CI. Furthermore, the Harbord regression-based test suggested no statistical evidence of small-study effects or publication bias (*P* = 0.901).

### Quality of the evidence

**Fig 5:**
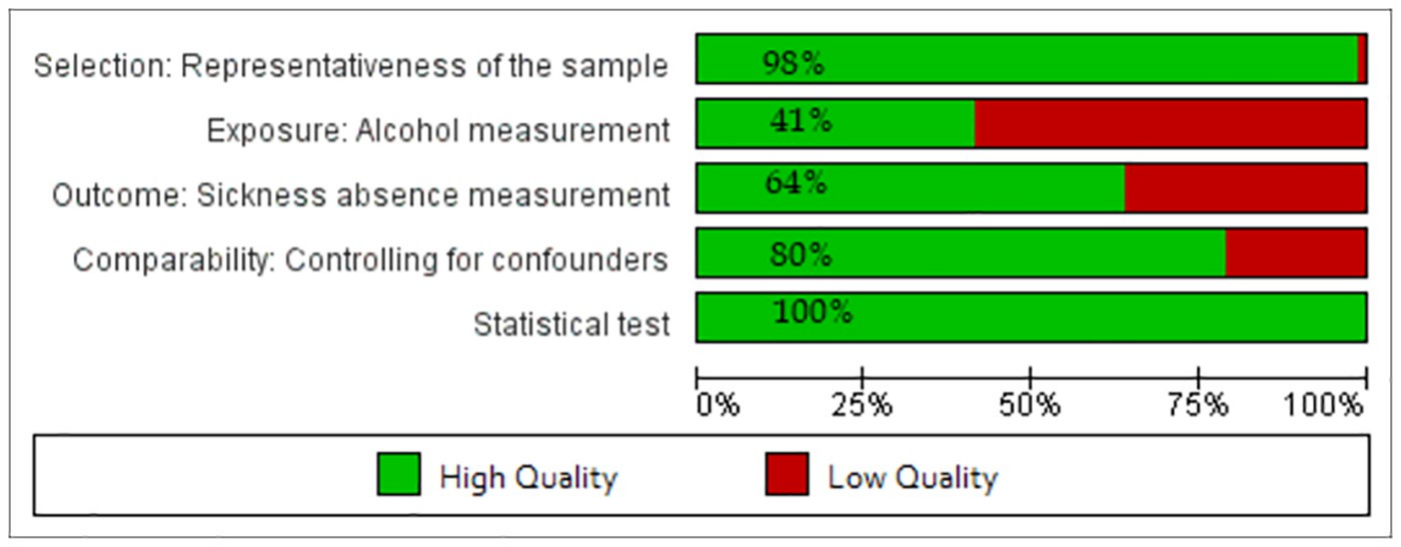
Quality of the associations on five key domains.

The quality assessment revealed that all the 162 tested associations had an adequate description of the statistical procedure, see Fig 5. Almost all of the (160 out of 162 (98%)) associations used probability or non-probability sampling techniques, and 41% of the associations (67 out of 162) measured alcohol using validated instruments such as AUDIT, or CDT blood test. About 57% of associations (38 of 67) using validated instruments and 59% of associations (56 of 95) using non-validated instruments were statistically significant. Around 64% of associations measured sickness absence by registry data (e.g., company or national registers), and the rest of them were self-reported absences. Among the 162 associations, 129 (80%) were adjusted for individual or/and environmental factors.

## Discussion

The aim of this systematic review and meta-analysis was to explore and uncover the relationship between alcohol use patterns and sickness absence by looking at differences in type of design (cross-sectional vs. longitudinal), type of data (self-reported vs. registered data), and type of sickness absence (long-term vs. short term). The following findings will be discussed: (i) revealed evidence for supporting a positive association between alcohol consumption patterns and sickness absence, (ii) high variability of measurements and study designs assessing alcohol consumption and sickness absence in the literature, and (iii) a diversity in social benefit and organizational factors, which might challenge generalization of the results in other countries and settings.

Both pooled estimates and descriptive evaluation, showed that higher levels of alcohol consumption are associated with higher levels of sickness absence, and that risky drinking patterns (as opposed to a low-risk pattern) are associated with a statistically significant increase in likelihood of sickness absence. These results are consistent with earlier reviews [39–41]. However, the results of the association between alcohol consumption, risky drinking and sickness absence in this review likely depend on a range of factors, one of which may be high variability of measurements and study designs assessing alcohol consumption and sickness absence.

In recent meta-analyses, Amiri and Behnezhad [40], as well as Marzan et al. [41] concluded that consuming alcohol constitutes a risk factor for sickness absence, but did not distinguish between short-term and long-term absences. In the current meta-analysis, the pooled estimates yielded a statistically significant association between risky drinking and short-term sickness absence, which might be explained by injury or hang-over one day absence [72]. Although, Schou and Moan [39] did not conduct a meta-analysis, they also found stronger support for the association between alcohol consumption and short-term absence than between alcohol consumption and long-term absence. While long-term sickness absence has been reported to be a better indicator of ill health than short-term absence [110, 111], being on long-term sickness absence was shown to reduce individuals’ alcohol consumption [83]. Moreover, it is likely that there is a broader range of potential causes of long-term absences, which may not hold true for short-term absences [39, 112, 113].

In their review, Schou and Moan [39], found positive associations between alcohol consumption and sickness absence from 28 studies, but the associations were mainly retrieved from cross-sectional data. In the current review, the vast majority of associations indicating positive and statistically significant results were based on longitudinal data (66 of 94, 70%), implying a possible causal relation between total alcohol consumption and sickness absence. The causal relations were also found in three of the included studies using time-series analyses [85, 90, 91]. However, from the pooled estimates considering risky versus low-risk drinking, only cross-sectional studies were able to find the risky drinking-sickness absence association.

One may assume that the cross-sectional study designs not only impede the establishment of causal inference but may also be influenced by the data measurements as they are mostly conducted on self-reported data. In the current meta-analysis, studies using cross-sectional design were mainly based on self-reported sickness absence data, which can be assumed to be less reliable [35]. However, although self-reported sickness absence, which is mostly short-term, is based on individual’s self-assessment, and registered/certified sickness absence (mostly long-term) is generally based on the general practitioner’s assessment, whether an individual asks for medical help depends on the individual’s own decision. Therefore, self-assessment of one’s health may affect a person’s evaluation about when seeking help for sickness absence is really needed, which in turn may influence employees’ absence type (self-reported and certified) and absence duration (short-term and long-term) [28], and may further influence the direction and significance of study designs.

Current meta-analysis found risky drinking-sickness absence association in studies using self-reported absence data, which can be explained by the above-mentioned notion. Moreover, since sickness absence was assessed differently when comparing risky and low-risk drinking (e.g., varying from ≥ 1 day [34] to ≥ 10 days [60]) throughout the included studies, this estimate does not provide details concerning the exact length of the sickness absence. Regarding the alcohol consumption and sickness absence in general, most of the samples in the review measured sickness absence by using registry data (103 of 162, 64%), and accordingly the percentages of significant associations were higher among samples using registry data than self-reported data (60% vs. 40%). Keeping administrative registries of sickness absence data is common in some countries, particularly in the Nordic countries, which offers the opportunity to easily access information and explore the association between alcohol and working populations in detail [61, 114].

Furthermore, between-country variation in sickness absence including benefits and often how the social health protection (SOCPRO) systems in each country are organized may influence the type and duration of sickness absence [42]. For instance, comparing two included Nordic countries, the likelihood of sickness absence was significantly higher for all studies conducted in Norway [90, 93, 95, 98, 102, 107, 108], compared to studies from Denmark [61, 62, 86]. These rates might be affected by the existing sickness absence benefit systems in each country. In Norway, for example, it is rarely possible to lay off an employee due to long-term sickness absence, while being absent for more than 120 days within a year in Denmark could lead to lay off. Therefore, in general, Norway reports a higher rate of long-term sickness absence and in contrast lower rate of short-term sickness absence than Denmark [27].

In addition, individuals’ decisions about drinking alcohol and whether to take sickness absence or attend work are influenced by systematic and organizational factors in the workplace [115]. Blum et al [72], Bacharach et al. [65], and Cunradi et al. [33] showed that the degree to which drinking alcohol may serve as a precursor of sickness absence, depends on a few key factors, one of which may be the existing relation between individuals and their supervisors and work-related stressors (e.g., job burnout). In these studies, risky drinking was more likely to be observed among employees who had conflicts with their co-workers and supervisors, or employees reported job burnout. One may assume that the potential for predicting sickness absence by alcohol consumption may be reduced among employees whose supervisors tend to focus on attendance. In this regard, such employees are more likely to resort to presenteeism rather than being absent, in order to avoid being labeled as a troubled worker [65, 72].

### Implications

Overall, evidence supports that higher levels of alcohol consumption and risky drinking may increase the likelihood of sickness absence. Research has shown that, as a policy implication, reducing per capita alcohol consumption results in a reduction in both the sickness absence costs, as well as the imposed economic costs for industries and societies [90].

Earlier research suggests that workplace interventions that target environmental (e.g., supportive work environment) and individual (e.g., alcohol skill training, and stress management) factors should be implemented, as they most likely will promote healthier lifestyles [33, 116–118]. Further research is needed for exploring whether other nuanced conditional factors (e.g., age, smoking, obesity, and work stress), which were measured unevenly across the included studies, can affect the direction of the association between alcohol consumption and sickness absence, as either a mediator or moderator. Moreover, to find out the causal inference between alcohol and sickness absence, research should review longitudinal designed studies using registered data. In addition, focusing on short-term sickness absence in efforts of reducing and preventing injuries and hang-over one-day alcohol-related sickness absence may be beneficial. Future research may be benefited from having abstainers as a reference group against moderate and risky drinkers as the most recent systematic review and meta-analysis has found a higher risk of sickness absence among both abstainers and heavy drinkers when compared to moderate drinkers [41].

### Strengths and limitations

The present study holds some strengths. A major strength was the search strategy which ensured an up-to-date selection and review of potential studies, up until June 2020. Furthermore, we were able to do subgroup analyses of the studies eligible for meta-analyses based on pertinent characteristics of the studies. This enabled a more fine-grained investigation into to accumulated research regarding alcohol consumption and sickness absence.

The present study also holds some limitations. First, studies published prior to 1980 were not included in this review. Although it is likely that studies pre-dating our inclusion period are few and potentially not relevant for the present-day association between alcohol consumption and sickness absence due to changes in alcohol culture at work, sickness absence policies, cultural aspects, and working life in general, this limitation should be borne in mind when interpreting our results. Second, our eligibility criteria may have introduced a bias related to which studies we included. The eligibility criteria chosen were based on our knowledge of the research field and present an effort to ensure some degree of comparability between the included studies. Regardless, the criteria chosen, and procedures followed are well-documented, which makes it possible to reproduce and critically assess each step of the review process. Third, included studies were based on self-reported alcohol use. There is evidence that individuals having risky drinking patterns tend to underreport their alcohol consumption or avoid participating in health surveys [119, 120]. Hence, the estimates may not reflect the real alcohol consumption of respondents in the included studies and the alcohol consumption measures are likely underestimated. However, there is a difference between measuring mere consumption and measuring risky drinking or potential alcohol-related problems. The latter is commonly measured by means of self-reported composite instruments (e.g., AUDIT) [121]. Such instruments take into account that the relationship between alcohol and health is multi-faceted, and their potential to screen alcohol consumption and related risks in primary care settings are well documented [122, 123]. Forth, although converting the alcohol drinking units were based on each study’s national guideline, the existing variations both in low-risk drinking guidelines and accepted standard drink among countries [17], may affect the definition of risky drinking, as well as prevention efforts. For example, while a standard drink is defined as 14 grams/day by the U.S. drinking guidelines, this amount is defined as 8 grams/day and 19.75 grams/day in the UK and Japan, respectively [17]. Fifth, the included studies used different operationalizations of sickness absence. Accordingly, some of the variations in the estimates may be affected by variations in sickness absence operationalization. Sixth, the studies included in meta-analysis were highly heterogeneous, precluding strong conclusions regarding the estimated association between alcohol consumption and sickness absence, and this is further emphasized in the sub-group analyses.

## Conclusion

Sickness absence is an important welfare scheme giving economical job security when sick, but also large consequences for employees. It is associated with a variety of occupational outcomes when related to alcohol consumption (e.g., economic loss, productivity loss, or a risk of exclusion from work). This systematic review and meta-analysis supported, but also challenged the available evidence regarding the association between alcohol consumption and sickness absence among employees. This study revealed how certain types of design, data, and type of sickness absence may produce different, and even large effects. Therefore, treating the association between alcohol use and sickness absence differently also on an individual level within workplace health promotion programs for reducing and controlling alcohol intake, as well as identifying and addressing individuals’ and work settings’ conditions may help in preventing different types of sickness absence targeting employees.

## Supporting information

S1 File PRISMA checklist. (DOC)

S1 Table Primary database search strategy (based on search in Medline). (DOCX)

S2 Table Overview of the association tests (n = 162) between alcohol consumption and sickness absence measures. (DOCX)

S1 Fig Pooled odds estimates and forest plots for sickness absence among risky drinking versus low-risk drinking employees, stratified by study design. (TIF)

S2 Fig Pooled odds estimates and forest plots for sickness absence among risky drinking versus low-risk drinking employees, stratified by sickness absence measures. (TIF)

S3 Fig Pooled odds estimates and forest plots for sickness absence among risky drinking versus low-risk drinking employees, stratified by sickness absence duration. (TIF)

S4 Fig Pooled odds estimates and forest plots for sickness absence among risky drinking versus low-risk drinking employees, stratified by year of publication. (TIF)

S5 Fig Pooled odds estimates and forest plots for sickness absence among risky drinking versus low-risk drinking employees, stratified by geographical region of the studies. (TIF)

## References

[1] WHO. World Health Organization (WHO). Global status report on alcohol and health. 2018.

[2] Thørrisen MM, Skogen JC, Aas RW. The associations between employees’ risky drinking and sociodemographics, and implications for intervention needs. BMC Public Health. 2018;18(1):735. doi: 10.1186/s12889-018-5660-x 29898703 PMC6000943

[3] Marchand A, Parent-Lamarche A, Blanc ME. Work and high-risk alcohol consumption in the Canadian workforce. Int J Environ Res Public Health. 2011;8(7):2692–705. doi: 10.3390/ijerph8072692 21845153 PMC3155324

[4] Babor TF, Higgins-Biddle JC, Saunders JB, Monteiro MG. The Alcohol Use Disorders Identification Test: Guidelines for use in primary care, WHO document WHO/MSD/MSB/01.6a. 2nd, editor. Geneva, Switzerland: World Health Organization; 2001.

[5] Harris MM, Heft LL. Alcohol and drug use in the workplace: Issues, controversies, and directions for future research. Journal of Management. 1992;.18(2):pp.

[6] Moore S, Grunberg L, Greenberg E. The relationships between alcohol problems and well-being, work attitudes, and performance: Are they monotonic? Journal of Substance Abuse. 2000;11(2):183–204. doi: 10.1016/s0899-3289(00)00020-1 10989778

[7] Mangione TW, Howland J, Amick B, Cote J, Lee M, Bell N, et al. Employee drinking practices and work performance. Journal Of Studies On Alcohol. 1999;60(2):261–70. doi: 10.15288/jsa.1999.60.261 10091965

[8] Sagvaag H, Rimstad SL, Kinn LG, Aas R. Six shades of grey: Identifying drinking culture and potentially risky drinking behaviour in the grey zone between work and leisure. The WIRUS culture study. Public Health Research. 2019;8(2). doi: 10.4081/jphr.2019.1585 31572696 PMC6747020

[9] Buvik K. It’s time for a drink! Alcohol as an investment in the work environment. Drug-Educ Prev Polic. 2020;27(1):86–91.

[10] Gordon R, Heim D, MacAskill S. Rethinking drinking cultures: a review of drinking cultures and a reconstructed dimensional approach. Public Health. 2012;126(1):3–11. doi: 10.1016/j.puhe.2011.09.014 22137093

[11] Thørrisen MM, Bonsaksen T, Hashemi N, Kjeken I, van Mechelen W, Aas RW. Association between alcohol consumption and impaired work performance (presenteeism): a systematic review. BMJ Open. 2019;9(7):e029184. doi: 10.1136/bmjopen-2019-029184 31315869 PMC6661906

[12] Moan IS, Halkjelsvik T. Socio-demographic differences in alcohol-related work impairment. Addiction (Abingdon, England). 2020. doi: 10.1111/add.15202 32707598

[13] Moan IS, Halkjelsvik T. Work-Related Alcohol Use and Harm to Others. Substance Use & Misuse. 2020:1–9. doi: 10.1080/10826084.2020.1801744 32804007

[14] Nielsen MB, Gjerstad J, Frone MR. Alcohol Use and Psychosocial Stressors in the Norwegian Workforce. Subst Use Misuse. 2017:1–11. doi: 10.1080/10826084.2017.1349797 28910176

[15] Cercarelli R, Allsop S, Evans M, Velander F. Reducing alcohol-related harm in the workplace: An evidence review—full report2012.

[16] International Alliance for Responsible Drinking, Drinking Guidelines: General Population, London, UK: International Journal for Responsible Drinking.; 2019.

[17] Dawson DA. Defining risk drinking. Alcohol Res Health. 2011;34(2):144–56. 22330212 PMC3860565

[18] Makela P, Bloomfield K, Gustafsson NK, Huhtanen P, Room R. Changes in volume of drinking after changes in alcohol taxes and travellers’ allowances: results from a panel study. Addiction. 2008;103(2):181–91. doi: 10.1111/j.1360-0443.2007.02049.x 18028522

[19] Heeb JL, Gmel G, Zurbrugg C, Kuo M, Rehm J. Changes in alcohol consumption following a reduction in the price of spirits: a natural experiment in Switzerland. Addiction. 2003;98(10):1433–46. doi: 10.1046/j.1360-0443.2003.00461.x 14519181

[20] Wagenaar AC, Tobler AL, Komro KA. Effects of Alcohol Tax and Price Policies on Morbidity and Mortality: A Systematic Review. American Journal of Public Health. 2010;100(11):2270–8. doi: 10.2105/AJPH.2009.186007 20864710 PMC2951962

[21] Kujala V, Tammelin T, Remes J, Vammavaara E, Ek E, Laitinen J. Work ability index of young employees and their sickness absence during the following year. Scandinavian Journal Of Work, Environment & Health. 2006;32(1):75–84. doi: 10.5271/sjweh.979 16539175

[22] Whitaker SC. The management of sickness absence. Occupational and Environmental Medicine. 2001;58(6):420–4. doi: 10.1136/oem.58.6.420 11351060 PMC1740146

[23] Folger J. The Causes and Costs of Absenteeism 2021 [https://www.investopedia.com/articles/personal-finance/070513/causes-and-costs-absenteeism.asp.

[24] European Foundation for the Improvement of Living and Working Conditions. Absence from work. [Internet]. 2010.

[25] Ruhle SA, Suss S. Presenteeism and Absenteeism at Work-an Analysis of Archetypes of Sickness Attendance Cultures. Journal of Business and Psychology. 2020;35(2):241–55.

[26] Jourdain G, Vezina M. How psychological stress in the workplace influences presenteeism propensity: A test of the Demand-Control-Support model. European Journal of Work and Organizational Psychology. 2014;23(4):483–96.

[27] Thorsen SV, Friborg C, Lundstrøm B, Kausto J, Örnelius K, Sundell T, et al. Sickness Absence in the Nordic Countries Nordic Social Statistical Committee (NOSOSCO); 2015.

[28] Hauge KE, Ulvestad M. Having a bad attitude? The relationship between attitudes and sickness absence. IZA Journal of Labor Policy. 2017;6:1–27.

[29] Dale-Olsen H, Markussen S. Økende sykefravær over tid?–Sykefravær, arbeid og trygd 1972–2008. Søkelys på arbeidslivet. 2010;27(1–02):105-.

[30] Buvik K, Moan IS, Halkjelsvik T. Alcohol-related absence and presenteeism: Beyond productivity loss. International Journal of Drug Policy. 2018;58:71–7. doi: 10.1016/j.drugpo.2018.05.005 29864644

[31] Grimsmo A, Rossow I.M. Alkohol og sykefravær(Alcohol and sickness absence). SIFA rapport 1997.

[32] Hammer T. Sykefravær og rusmiddelbruk blant unge i arbeid(Sickness absence and misuse of drugs among young people in work). NOVA rapport; 1999.

[33] Cunradi CB, Greiner BA, Ragland DR, Fisher J. Alcohol, stress-related factors, and short-term absenteeism among urban transit operators. J Urban Health. 2005;82(1):43–57. doi: 10.1093/jurban/jti007 15738336 PMC3456620

[34] Roche AM, Pidd K, Berry JG, Harrison JE. Workers’ drinking patterns: the impact on absenteeism in the Australian work-place. Addiction. 2008;103(5):738–48. doi: 10.1111/j.1360-0443.2008.02154.x 18412752

[35] Stapelfeldt CM, Jensen C, Andersen NT, Fleten N, Nielsen CV. Validation of sick leave measures: self-reported sick leave and sickness benefit data from a Danish national register compared to multiple workplace-registered sick leave spells in a Danish municipality. Bmc Public Health. 2012;12. 10.1186/1471-2458-12-661 PMC3511193 22894644

[36] Kivimäki M, Vahtera J, Elovainio M, Lillrank B, Kevin MV. Death or illness of a family member, violence, interpersonal conflict, and financial difficulties as predictors of sickness absence: longitudinal cohort study on psychological and behavioral links. Psychosomatic Medicine. 2002;64(5):817–25. 12271113

[37] Vahtera J, Poikolainen K, Kivimäki M, Ala-Mursula L, Pentti J. Alcohol intake and sickness absence: a curvilinear relation. American Journal Of Epidemiology. 2002;156(10):969–76. doi: 10.1093/aje/kwf138 12419770

[38] Marmot M, Feeney A, Shipley M, North F, Syme SL. Sickness Absence as a Measure of Health-Status and Functioning—from the Uk Whitehall-Ii Study. J Epidemiol Commun H. 1995;49(2):124–30. doi: 10.1136/jech.49.2.124 7798038 PMC1060095

[39] Schou L, Moan IS. Alcohol use-sickness absence association and the moderating role of gender and socioeconomic status: A literature review. Drug Alcohol Rev. 2016;35(2):158–69. doi: 10.1111/dar.12278 26331574

[40] Amiri S, Behnezhad S. Alcohol consumption and sick leave: a meta-analysis. Journal of Addictive Diseases. 2020;38(2):100–12. doi: 10.1080/10550887.2020.1724606 32037988

[41] Marzan M, Callinan S, Livingston M, Leggat G, Jiang H. Systematic Review and Dose–Response Meta-Analysis on the Relationship Between Alcohol Consumption and Sickness Absence. Alcohol Alcoholism. 2021. doi: 10.1093/alcalc/agab008 33604615

[42] Scheil-Adlung X, Sandner L. The case for paid sick leave (World Health Report). World Health Organization (WHO); 2010.

[43] Schünemann HJ, Oxman AD, Vist GE, Higgins JPT, Deeks JJ, Glasziou P. Interpreting results and drawing conclusions. In: Higgins JPT, Green S, eds. Cochrane handbook for systematic reviews of interventions. 2008.

[44] Chien PF, Khan KS, Siassakos D. Registration of systematic reviews: PROSPERO. BJOG. 2012;119(8):903–5. doi: 10.1111/j.1471-0528.2011.03242.x 22703418

[45] Moher D, Liberati A, Tetzlaff J, Altman DG, Group P. Preferred reporting items for systematic reviews and meta-analyses: the PRISMA statement. BMJ. 2009;339:b2535. doi: 10.1136/bmj.b2535 19622551 PMC2714657

[46] Drummond C, Hillyard M, Leonhardt M, Wurst F, Dom G, Mann K, et al. Comparison of European Clinical Guidelines on the Management of Alcohol Use Disorders. European Addiction Research. 2020. doi: 10.1159/000512112 33291106

[47] Wang YM, Zhou QY, Zhu JZ, Zhu KF, Yu CH, Li YM. Systematic Review with Meta-Analysis: Alcohol Consumption and Risk of Colorectal Serrated Polyp. Dig Dis Sci. 2015;60(7):1889–902. doi: 10.1007/s10620-014-3518-3 25618311

[48] Anderer P, Møller L, Galea C. Alcohol in the European Union; Consumption, harm and policy approaches. Denmark: World Health Organization (WHO); 2012.

[49] Modesti PA, Reboldi G, Cappuccio FP, Agyemang C, Remuzzi G, Rapi S, et al. Panethnic Differences in Blood Pressure in Europe: A Systematic Review and Meta-Analysis. Plos One. 2016;11(1). doi: 10.1371/journal.pone.0147601 26808317 PMC4725677

[50] Wells G, Shea B, O’Connell D, Peterson J, Welch V, Losos M, et al. The Newcastle-Ottawa Scale (NOS) for assessing the quality of nonrandomised studies in meta-analyses2013; (http://www.ohri.ca/programs/clinical_epidemiology/oxford.asp).

[51] Higgins JPT, Thompson SG, Deeks JJ, Altman DG. Measuring inconsistency in meta-analyses. Brit Med J. 2003;327(7414):557–60. doi: 10.1136/bmj.327.7414.557 12958120 PMC192859

[52] L’Abbe KA, Detsky AS, O’Rourke K. Meta-analysis in clinical research. Ann Intern Med. 1987;107(2):224–33. doi: 10.7326/0003-4819-107-2-224 3300460

[53] Higgins JPT, Thompson SG. Quantifying heterogeneity in a meta-analysis. Stat Med. 2002;21(11):1539–58. doi: 10.1002/sim.1186 12111919

[54] Harbord RM, Egger M, Sterne JA. A modified test for small-study effects in meta-analyses of controlled trials with binary endpoints. Stat Med. 2006;25(20):3443–57. doi: 10.1002/sim.2380 16345038

[55] StataCorp. 2019. Stata Statistical Software: Release 16. College Station, TX: StataCorp LLC. [Internet].

[56] Hermansson U, Helander A, Brandt L, Huss A, Ronnberg S. The alcohol use disorders identification test and carbohydrate-deficient transferrin in alcohol-related sickness absence. Alcoholism: Clinical and Experimental Research. 2002;26(1):28–35. 11821651

[57] Kondo K, Kobayashi Y, Hirokawa K, Tsutsumi A, Kobayashi F, Haratani T, et al. Job strain and sick leave among Japanese employees: a longitudinal study. Int Arch Occ Env Hea. 2006;79(3):213–9. doi: 10.1007/s00420-005-0027-x 16283366

[58] Laaksonen M, Piha K, Martikainen P, Rahkonen O, Lahelma E. Health-related behaviours and sickness absence from work. Occupational And Environmental Medicine. 2009;66(12):840–7. doi: 10.1136/oem.2008.039248 19934118

[59] Hensing G, Holmgren K, Mårdby AC. Harmful alcohol habits were no more common in a sample of newly sick-listed Swedish women and men compared with a random population sample. Alcohol And Alcoholism (Oxford, Oxfordshire). 2011;46(4):471–7. 10.1093/alcalc/agr033 21486930

[60] Kaila-Kangas L, Koskinen A, Leino-Arjas P, Virtanen M, Härkänen T, Lallukka T. Alcohol use and sickness absence due to all causes and mental- or musculoskeletal disorders: a nationally representative study. BMC Public Health. 2018;18(1):152-. doi: 10.1186/s12889-018-5059-8 29343233 PMC5773150

[61] Jørgensen MB, Thygesen LC, Becker U, Tolstrup JS. Alcohol consumption and risk of unemployment, sickness absence and disability pension in Denmark: a prospective cohort study. Addiction (Abingdon, England). 2017;112(10):1754–64. doi: 10.1111/add.13875 28544338

[62] Jorgensen MB, Pedersen J, Thygesen LC, Lau CJ, Christensen AI, Becker U, et al. Alcohol consumption and labour market participation: a prospective cohort study of transitions between work, unemployment, sickness absence, and social benefits. Eur J Epidemiol. 2019. 10.1007/s10654-018-0476-7 PMC6451700 30627937

[63] Richmond RL, Kehoe L, Hailstone S, Wodak A, Uebel-Yan M. Quantitative and qualitative evaluations of brief interventions to change excessive drinking, smoking and stress in the police force. Addiction (Abingdon, England). 1999;94(10):1509–21. doi: 10.1046/j.1360-0443.1999.941015097.x 10790903

[64] Ovuga E, Madrama C. Burden of alcohol use in the Uganda Police in Kampala District. African Health Sciences. 2006;6(1):14–20. doi: 10.5555/afhs.2006.6.1.14 16615821 PMC1831968

[65] Bacharach SB, Bamberger P, Biron M, Bacharach SB, Bamberger P, Biron M. Alcohol consumption and workplace absenteeism: the moderating effect of social support. Journal of Applied Psychology. 2010;95(2):334–48. doi: 10.1037/a0018018 20230073 PMC2903009

[66] Chakraborty S, Subramanya AHC. Socio-demographic and clinical predictors of absenteeism—A cross-sectional study of urban industrial employees. Industrial Psychiatry Journal. 2013;22(1):17–21. doi: 10.4103/0972-6748.123589 24459368 PMC3895306

[67] Mekonnen TH, Lamessa SK, Wami SD. Sickness-related absenteeism and risk factors associated among flower farm industry workers in Bishoftu town, Southeast Ethiopia, 2018: a cross-sectional study. BMC research notes. 2019;12(1):181. doi: 10.1186/s13104-019-4223-2 30922369 PMC6440003

[68] Jenkins R. Sex differences in alcohol consumption and its associated morbidity in young civil servants. Brit J Addict. 1986;81(4):525–35. doi: 10.1111/j.1360-0443.1986.tb00364.x 3463352

[69] Persson J, Magnusson PH. Sickness absenteeism and mortality in patients with excessive drinking in somatic out-patient care. Scand J Prim Health Care. 1989;7(4):211–7. doi: 10.3109/02813438909088666 2533993

[70] Marmot MG, North F, Feeney A, Head J. Alcohol consumption and sickness absence: from the Whitehall II study. Addiction (Abingdon, England). 1993;88(3):369–82. doi: 10.1111/j.1360-0443.1993.tb00824.x 8461854

[71] North F, Syme SL, Feeney A, Head J, Shipley MJ, Marmot MG. Explaining socioeconomic differences in sickness absence: the Whitehall II Study. BMJ (Clinical Research Ed). 1993;306(6874):361–6. doi: 10.1136/bmj.306.6874.361 8461681 PMC1676477

[72] Blum TC, Roman PM, Martin JK. Alcohol consumption and work performance. Journal Of Studies On Alcohol. 1993;54(1):61–70. doi: 10.15288/jsa.1993.54.61 8355501

[73] French MT, Zarkin GA, Hartwell TD, Bray JW. Prevalence and consequences of smoking, alcohol use, and illicit drug use at five worksites. Public Health Reports (Washington, DC: 1974). 1995;110(5):593–9. PMC1381637 7480614

[74] Vasse RM, Nijhuis FJN, Kok G. Associations between work stress, alcohol consumption and sickness absence. Addiction. 1998;93(2):231–41. doi: 10.1046/j.1360-0443.1998.9322317.x 9624724

[75] Spak F, Hensing G, Allebeck P. Sick-leave in women with alcohol dependence or abuse: effects of additional psychiatric disorders. Social Psychiatry And Psychiatric Epidemiology. 1998;33(12):613–9. doi: 10.1007/s001270050101 9857794

[76] Upmark M, Möller J, Romelsjö A. Longitudinal, population-based study of self reported alcohol habits, high levels of sickness absence, and disability pensions. J Epidemiol Commun H. 1999;53(4):223–9. 10.1136/jech.53.4.223 PMC1756858 10396548

[77] Upmark M, Karlsson G, Romelsjö A. Drink driving and criminal behaviours as risk factors for receipt of disability pension and sick leave: a prospective study of young men. Addiction (Abingdon, England). 1999;94(4):507–19. doi: 10.1046/j.1360-0443.1999.9445076.x 10605847

[78] Holder HD, Blose JO. A comparison of occupational and nonoccupational disability payments and work absences for alcoholics and nonalcoholics. J Occup Med. 1991;33(4):453–7. 1828080

[79] McFarlin SK, Fals-Stewart W. Workplace absenteeism and alcohol use: A sequential analysis. Psychology of Addictive Behaviors. 2002;16(1):17–21. doi: 10.1037//0893-164x.16.1.17 11934081

[80] Bendtsen P, Hensing G, Alexanderson K. Self-perceived excessive alcohol consumption among employed women: Association with health and psychosocial factors. Addict Behav. 2003;28(4):777–83. doi: 10.1016/s0306-4603(01)00294-5 12726790

[81] Morikawa Y, Martikainen P, Head J, Marmot M, Ishizaki M, Nakagawa H. A comparison of socio-economic differences in long-term sickness absence in a Japanese cohort and a British cohort of employed men. European Journal Of Public Health. 2004;14(4):413–6. doi: 10.1093/eurpub/14.4.413 15542879

[82] Voss M, Floderus B, Diderichsen F. How do job characteristics, family situation, domestic work, and lifestyle factors relate to sickness absence? A study based on Sweden Post. J Occup Environ Med. 2004;46(11):1134–43. doi: 10.1097/01.jom.0000145433.65697.8d 15534500

[83] Floderus B, Göransson S, Alexanderson K, Aronsson G. Self-estimated life situation in patients on long-term sick leave. J Rehabil Med. 2005;37(5):291–9. doi: 10.1080/16501970510034422 16203618

[84] Pidd KJ, Berry JG, Roche AM, Harrison JE. Estimating the cost of alcohol-related absenteeism in the Australian workforce: The importance of consumption patterns. The Medical Journal Of Australia. 2006;185(11–12):637–41. doi: 10.5694/j.1326-5377.2006.tb00738.x 17181511

[85] Norstrom T. Per capita alcohol consumption and sickness absence. Addiction. 2006;101(10):1421–7. doi: 10.1111/j.1360-0443.2006.01446.x 16968343

[86] Christensen KB, Lund T, Labriola M, Bültmann U, Villadsen E. The impact of health behaviour on long term sickness absence: results from DWECS/DREAM. Industrial Health. 2007;45(2):348–51. doi: 10.2486/indhealth.45.348 17485882

[87] Suominen S, Vahtera J, Korkeila K, Helenius H, Kivimäki M, Koskenvuo M. Job strain, life events, and sickness absence: a longitudinal cohort study in a random population sample. J Occup Environ Med. 2007;49(9):990–6. doi: 10.1097/JOM.0b013e3181343e2b 17848855

[88] Johansson E, Bockerman P, Uutela A. Alcohol consumption and sickness absence: Evidence from microdata. European Journal of Public Health. 2009;19(1):19–22. doi: 10.1093/eurpub/ckn116 19033355

[89] Salonsalmi A, Laaksonen M, Lahelma E, Rahkonen O. Drinking habits and sickness absence: the contribution of working conditions. Scand J Public Health. 2009;37(8):846–54. doi: 10.1177/1403494809350519 19828773

[90] Norstrom T, Moan IS. Per capita alcohol consumption and sickness absence in Norway. Eur J Public Health. 2009;19(4):383–8. doi: 10.1093/eurpub/ckp044 19369492

[91] Balsa AI, French MT. ALCOHOL USE AND THE LABOR MARKET IN URUGUAY. Health Economics. 2010;19(7):833–54. doi: 10.1002/hec.1520 19548325

[92] Kirkham HS, Clark BL, Bolas CA, Lewis GH, Jackson AS, Fisher D, et al. Which modifiable health risks are associated with changes in productivity costs? Population Health Management. 2015;18(1):30–8. doi: 10.1089/pop.2014.0033 25375893

[93] Edvardsen HME, Moan IS, Christophersen AS, Gjerde H. Use of alcohol and drugs by employees in selected business areas in Norway: a study using oral fluid testing and questionnaires. J Occup Med Toxicol. 2015;10. doi: 10.1186/s12995-015-0087-0 26681976 PMC4682215

[94] Lidwall U, Marklund S. Trends in long-term sickness absence in Sweden 1992–2008: the role of economic conditions, legislation, demography, work environment and alcohol consumption. Int J Soc Welf. 2011;20(2):167–79.

[95] Schou L, Storvoll EE, Moan IS. Alcohol-related sickness absence among young employees: gender differences and the prevention paradox. Eur J Public Health. 2014;24(3):480–5. doi: 10.1093/eurpub/cku035 24675063

[96] Ervasti J, Kivimäki M, Head J, Goldberg M, Airagnes G, Pentti J, et al. Sociodemographic Differences Between Alcohol Use and Sickness Absence: Pooled Analysis of Four Cohort Studies. Alcohol And Alcoholism (Oxford, Oxfordshire). 2018;53(1):95–103. doi: 10.1093/alcalc/agx079 29040353

[97] Ervasti J, Kivimaki M, Head J, Goldberg M, Airagnes G, Pentti J, et al. Sickness absence diagnoses among abstainers, low-risk drinkers and at-risk drinkers: consideration of the U-shaped association between alcohol use and sickness absence in four cohort studies. Addiction (Abingdon, England). 2018. doi: 10.1111/add.14249 29873143 PMC6099368

[98] Torvik FA, Reichborn-Kjennerud T, Gjerde LC, Knudsen GP, Ystrom E, Tambs K, et al. Mood, anxiety, and alcohol use disorders and later cause-specific sick leave in young adult employees. Bmc Public Health. 2016;16. 10.1186/s12889-016-3427-9 PMC4972995 27488425

[99] Silva-Junior JSd, Fischer FM. Long-term sickness absence due to mental disorders is associated with individual features and psychosocial work conditions. Plos One. 2014;9(12):e115885–e. doi: 10.1371/journal.pone.0115885 25531900 PMC4274157

[100] Richmond MK, Pampel FC, Wood RC, Nunes AP. Impact of Employee Assistance Services on Depression, Anxiety, and Risky Alcohol Use: A Quasi-Experimental Study. J Occup Environ Med. 2016;58(7):641–50. doi: 10.1097/JOM.0000000000000744 27389792

[101] De Clercq B, Clays E, Janssens H, De Bacquer D, Casini A, Kittel F, et al. Health Behaviours As a Mechanism in the Prospective Relation between Workplace Reciprocity and Absenteeism: A Bridge too Far? Plos One. 2015;10(11):e0141608–e. doi: 10.1371/journal.pone.0141608 26524011 PMC4629877

[102] Østby KA, Czajkowski N, Knudsen GP, Ystrøm E, Gjerde LC, Kendler KS, et al. Does low alcohol use increase the risk of sickness absence? A discordant twin study. BMC Public Health. 2016;16(1):825-. doi: 10.1186/s12889-016-3502-2 27538396 PMC4990980

[103] Morois S, Airagnes G, Lemogne C, Leclerc A, Limosin F, Goldberg S, et al. Daily alcohol consumption and sickness absence in the GAZEL cohort. Eur J Public Health. 2017;27(3):482–8. doi: 10.1093/eurpub/ckx012 28339654

[104] Ervasti J, Kivimaki M, Pentti J, Halonen JI, Vahtera J, Virtanen M. Changes in drinking as predictors of changes in sickness absence: a case-crossover study. J Epidemiol Commun H. 2018;72(1):61–7. doi: 10.1136/jech-2017-209777 29101213

[105] Salonsalmi A, Rahkonen O, Lahelma E, Laaksonen M. Changes in alcohol drinking and subsequent sickness absence. Scand J Public Healt. 2015;43(4):364–72. doi: 10.1177/1403494815574154 25743874

[106] Araujo MYC, Sarti FM, Fernandes RA, Monteiro HL, Turi BC, Anokye N, et al. Association Between Costs Related to Productivity Loss and Modified Risk Factors Among Users of the Brazilian National Health System. J Occup Environ Med. 2017;59(3):313–9. doi: 10.1097/JOM.0000000000000951 28267102

[107] Schou L, Birkelund GE. Alcohol-related sickness absence of young employees in Norway: The impact of social roles and socioeconomic status. Nord Stud Alcohol Dr. 2015;32(4):411–26.

[108] Lund I, Moan IS, Edvardsen HME. The relative impact of smoking, alcohol use and drug use on general sickness absence among Norwegian employees. BMC Public Health. 2019;19(1):N.PAG–N.PAG. doi: 10.1186/s12889-019-6891-1 31053139 PMC6499980

[109] Landberg J, Hemmingsson T, Syden L, Ramstedt M. The Contribution of Alcohol Use, Other Lifestyle Factors and Working Conditions to Socioeconomic Differences in Sickness Absence. European Addiction Research. 2020;26(1):40–51. doi: 10.1159/000504437 31747671 PMC6979426

[110] Kivimaki M, Head J, Ferrie JE, Shipley MJ, Vahtera J, Marmot MG. Sickness absence as a global measure of health: evidence from mortality in the Whitehall II prospective cohort study. Brit Med J. 2003;327(7411):364–8. doi: 10.1136/bmj.327.7411.364 12919985 PMC175810

[111] Vahtera J, Pentti J, Kivimäki M. Sickness absence as a predictor of mortality among male and female employees. J Epidemiol Commun H. 2004;58(4):321–6. doi: 10.1136/jech.2003.011817 15026447 PMC1732735

[112] Udo T, Vasquez E, Shaw BA. A lifetime history of alcohol use disorder increases risk for chronic medical conditions after stable remission. Drug Alcohol Depen. 2015;157:68–74. doi: 10.1016/j.drugalcdep.2015.10.008 26482092

[113] Odlaug BL, Gual A, DeCourcy J, Perry R, Pike J, Heron L, et al. Alcohol Dependence, Co-occurring Conditions and Attributable Burden. Alcohol Alcoholism. 2016;51(2):201–9. doi: 10.1093/alcalc/agv088 26246514 PMC4755551

[114] Hjollund NH, Larsen FB, Andersen JH. Register-based follow-up of social benefits and other transfer payments: Accuracy and degree of completeness in a Danish interdepartmental administrative database compared with a population-based survey. Scand J Public Healt. 2007;35(5):497–502. 10.1080/14034940701271882 17852980

[115] Johansson G, Lundberg I, Marklund S, Bjurvald M, Hogstedt C, Palmer E, et al. Sjukflexibilitetsmodellen- utgångspunkter og resultat [The disease flexibility model—starting points and results]. Stockholm: Arbeidslivsinstituttet; 2005.

[116] Ragland DR, Krause N, Greiner BA, Fisher JM. Studies of health outcomes in transit operators: policy implications of the current scientific database. J Occup Health Psychol. 1998;3(2):172–87. doi: 10.1037//1076-8998.3.2.172 9585916

[117] Kivlahan DR, Marlatt GA, Fromme K, Coppel DB, Williams E. Secondary Prevention with College Drinkers—Evaluation of an Alcohol Skills Training-Program. Journal of Consulting and Clinical Psychology. 1990;58(6):805–10. doi: 10.1037//0022-006x.58.6.805 2292630

[118] Landau JC. The Impact of a Change in an Attendance Control-System on Absenteeism and Tardiness (Vol 13, 1994). Journal of Organizational Behavior Management. 1994;14(2):U104–U6.

[119] Boniface S, Scholes S, Shelton N, Connor J. Assessment of Non-Response Bias in Estimates of Alcohol Consumption: Applying the Continuum of Resistance Model in a General Population Survey in England. PLoS One. 2017;12(1):e0170892. doi: 10.1371/journal.pone.0170892 28141834 PMC5283659

[120] Knudsen AK, Hotopf M, Skogen JC, Overland S, Mykletun A. The Health Status of Nonparticipants in a Population-based Health Study The Hordaland Health Study. American Journal of Epidemiology. 2010;172(11):1306–14. doi: 10.1093/aje/kwq257 20843863

[121] Skogen JC, Thorrisen MM, Olsen E, Hesse M, Aas RW. Evidence for essential unidimensionality of AUDIT and measurement invariance across gender, age and education. Results from the WIRUS study. Drug Alcohol Depend. 2019;202:87–92. doi: 10.1016/j.drugalcdep.2019.06.002 31325821

[122] Hays RD, Merz JF, Nicholas R. Response Burden, Reliability, and Validity of the Cage, Short Mast, and Audit Alcohol Screening Measures. Behav Res Meth Instr. 1995;27(2):277–80.

[123] Conigrave KM, Saunders JB, Reznik RB. Predictive Capacity of the Audit Questionnaire for Alcohol-Related Harm. Addiction. 1995;90(11):1479–85. doi: 10.1046/j.1360-0443.1995.901114796.x 8528033

