# Supplementary material for: A systematic review and meta-analysis uncovering the relationship between alcohol consumption and sickness absence. When type of design, data, and sickness absence make a difference
Source: PLoS One. 2022 Jan 11;17(1):e0262458. doi: 10.1371/journal.pone.0262458 (PMC8752011; doi:10.1371/journal.pone.0262458)
Supplement: S1 Table — (DOCX) [file pone.0262458.s002.docx]

**S2 Table.** Primary database search strategy (based on search in Medline)

|  | **Thematic blocks** | **Search#** | **Query** | **Search type** | **Search level** |
| --- | --- | --- | --- | --- | --- |
| Exposure | *Alcohol consumption* | 1 | alcohol* | Text | Abstract |
|  |  | 2 | drink* | Text | Abstract |
|  |  | 3 | drunk* | Text | Abstract |
|  |  | 4 | hangover | Text | Abstract |
|  |  | 5 | "hang over" | Text | Abstract |
|  |  | 6 | alcohol drinking | MeSH | - |
|  |  | 7 | binge drinking | MeSH | - |
|  |  | **8** | **1 OR 2 OR 3 OR 4 OR 5 OR 6 OR 7** | | |
| Outcome | *Sickness absence* | 9 | “sick leave” | Text | Abstract |
|  |  | 10 | “sickness absence” | Text | Abstract |
|  |  | 11 | absenteeism | Text | Abstract |
|  |  | 12 | “lost work days” | Text | Abstract |
|  |  | 13 | “lost work hours” | Text | Abstract |
|  |  | 14 | “leave of absence” | Text | Abstract |
|  |  | 15 | “work absence” | Text | Abstract |
|  |  | 16 | “illness days” | Text | Abstract |
|  |  | 17 | absenteeism | MeSH | - |
|  |  | 18 | sickness absence | MeSH | - |
|  |  | 19 | sick leave | MeSH | - |
|  |  | **20** | **9 OR 10 OR 11 OR 12 OR 13 OR 14 OR 15 OR 16 OR 17 OR 18 OR 19** | | |
|  |  | **21** | **8 AND 20** | | |
| *Note.* This primary database search strategy was applied in Medline. When applied in the other databases (Embase, Cinahl, PsycInfo, AMED, and Web of Science), the strategy was adapted to each database. | | | | | |
