# Supplementary material for: A systematic review and meta-analysis uncovering the relationship between alcohol consumption and sickness absence. When type of design, data, and sickness absence make a difference
Source: PLoS One. 2022 Jan 11;17(1):e0262458. doi: 10.1371/journal.pone.0262458 (PMC8752011; doi:10.1371/journal.pone.0262458)
Supplement: S2 Table — (DOCX) [file pone.0262458.s003.docx]

**S3 Table. Overview of the association tests (n=162) between alcohol consumption and sickness absence measures**

| **Association ID** | **Study** | **Gender** | **Significance**  **P-value/CI** | **Direction**  **P/N ^a^** | **Adjustment** | **Sickness absence measure ^b^** |
| --- | --- | --- | --- | --- | --- | --- |
| 1 | Jenkins (1986) | Both | *p* <.005 | P | No | ND |
| 2 | Persson & Magnusson (1989) | Both | *p* <.001 | P | No | ND |
| 3 | " | Both | *p* <.001 | P | No | ND |
| 4 | Marmot et al. (1993) | Male | CI: 0.97-1.12 | P | Age and grade of employment | S |
| 5 | " | Female | CI: 0.84-1.06 | N | Age and grade of employment | S |
| 6 | " | Male | CI: 0.96-1.30 | P | Age and grade of employment | L |
| 7 | " | Female | CI: 0.60-1.03 | N | Age and grade of employment | L |
| 8 | North et al. (1993) | Male | CI: 1.58-2.20 | P | Age, smoking, frequency of alcohol consumption, work characteristics, social circumstances outside work and demographic factors | S |
| 9 | " | Female | CI: 1.13-2.75 | P | '' | S |
| 10 | " | Male | CI: 1.46-3.07 | P | '' | L |
| 11 | " | Female | CI: 0.51-2.90 | P | '' | L |
| 12 | Blum (1993) | Male | *p* > .05 | P | No | ND |
| 13 | " | Male | *p* < .01 | P | No | ND |
| 14 | " | Male | *p* < .05 | P | No | ND |
| 15 | French et al. (1995) | Both | *p* < .05 | P | No | ND |
| 16 | Vasse et al. (1998) | Both | *p* > .05 | N | Socio-demographic variables, smoking and work stressors | ND |
| 17 | " | Both | *p* > 0.10 | P | '' | ND |
| 18 | Spak et al. (1998) | Female | CI: 6.32-33.25 | P | No | ND |
| 19 | " | Female | *P* < 0.01 | P | Socio-economic | ND |
| 20 | " | Female | *P* < 0.01 | P | Socio-economic | ND |
| 21 | Upmark et al. (1999) | Male | CI: 0.5-1.9 | P | Age, socioeconomic status, smoking habits, and self-rated health | ND |
| 22 | " | Male | CI: 1.4-5.4 | P | '' | ND |
| 23 | " | Female | CI: 0.6-1.8 | P | '' | ND |
| 24 | " | Female | CI: 0.7-4.5 | P | '' | ND |
| 25 | " | Male | CI: 0.0-1.6 | N | '' | ND |
| 26 | " | Male | CI: 0.8-4.3 | P | '' | ND |
| 27 | " | Female | CI: 0.4-3.0 | P | '' | ND |
| 28 | " | Female | CI: 0.6-12.9 | P | '' | ND |
| 29 | Upmark et al. (2) (1999) | Male | CI: 2.0-2.8 | P | Psychosocial factors from conscription and for criminality | ND |
| 30 | " | Male | CI: 1.2-2.2 | P | No | ND |
| 31 | " | Male | CI: 0.9-1.5 | P | No | ND |
| 32 | Richmond et al. (1999) | Both | *p* ˂0.05 | P | No | ND |
| 33 | " | Both | *p* ˂0.05 | P | No | ND |
| 34 | Holder and Blose (1991) | Both | *p* < 0.001 | P | No | ND |
| 35 | Vahtera et al. (2002) | Both | *p* <0.001 | P | Socio-demographics, behavioral and biologic risk factors, psychosocial risk factors, and cardiovascular diseases | L |
| 36 | Hermansson et al. (2002) | Both | *p* = 0.047 | P | No | L |
| 37 | " | Both | *p* = 0.85 | P | No | L |
| 38 | " | Both | *p* = 0.15 | P | No | L |
| 39 | McFarlin & Fals-Stewart (2002) | Male | *p* > .05 | P | Socio-demographic and background variables, and worksite | ND |
| 40 | " | Male | *p* <.01 | P | '' | ND |
| 41 | " | Male | *p* > .05 | P | '' | ND |
| 42 | Kivimäki et al. (2002) | Male | ns | P | No | ND |
| 43 | " | Female | ns | N | No | ND |
| 44 | " | Male | *p* <.001 | P | No | ND |
| 45 | " | Female | ns | P | No | ND |
| 46 | Bendtsen et al. (2003) | Female | CI: 0.46-1.71 | P | Age | L |
| 47 | " | Female | CI: 1.31-3.61 | P | Age | L |
| 48 | " | Female | CI: 1.39-3.07 | P | Age | L |
| 49 | Morikawa et al. (2004) | Male | CI: 0.82-1.35 | P | Age | L |
| 50 | " | Male | CI: 0.69-0.84 | P | '' | L |
| 51 | " | Male | CI: 0.78-1.19 | P | '' | L |
| 52 | " | Male | CI: 0.65-0.80 | P | '' | L |
| 53 | Voss et al. (2004) | Male | CI: 1.5-3.8 | P | Age | ND |
| 54 | " | Female | CI: 0.5-2.8 | P | Age | ND |
| 55 | Cunradi et al. (2005) | Both | *p* < .01 | P | Age, gender, race/ethnicity, marital status, income, education, and seniority | S |
| 56 | " | Both | CI: 0.56-3.15 | P | '' | S |
| 57 | " | Both | *p* < .05 | P | '' | S |
| 58 | " | Both | *p* < .01 | P | '' | S |
| 59 | Floderus et al. (2005) | Both | CI: 0.54-1.36 | N | Gender and sick leave diagnosis | L |
| 60 | Ovuga & Madrama (2006) | Both | CI: 0.7-12.5 | P | No | ND |
| 61 | " | Both | CI: 1.02-12.00 | P | No | ND |
| 62 | Pidd et al. (2006) | Both | CI: 0.25-0.44 | P | Age and gender | S |
| 63 | " | Both | CI: 0.25-0.44 | P | Age and gender | L |
| 64 | Kondo et al. (2006) | Both | CI: 0.14-1.23 | N | Gender, age, education completed, occupation, and daily tobacco consumption, | L |
| 65 | " | Both | CI: 0.23-1.95 | N | '' | L |
| 66 | Kujala et al. (2006) | Male | CI: 0.46-1.67 | N | Work-related, family structure, lifestyle, living condition, and behavior characteristics | L |
| 67 | " | Female | CI: 0.53-1.55 | N | '' | ND |
| 68 | Norstrom (2006) | Male | *p* <0.05 | P | No | ND |
| 69 | " | Female | ns | P | No | ND |
| 70 | Christensen et al. (2007) | Male | CI: 0.85-1.98 | P | Family status, socio economic status, education, and diagnosed disease | L |
| 71 | " | Female | CI: 0.76-1.83 | P | '' | L |
| 72 | Suominen et al. (2007) | Both | CI: 0.72-1.20 | N | Age, socioeconomic status | L |
| 73 | Johansson et al. (2009) | Both | *p* <.001 | P | Age, gender, marital status, education | ND |
| 74 | Laaksonen et al. (2009) | Male | CI: 1.01-1.55 | P | Age | S |
| 75 | " | Female | CI: 1.06-1.26 | P | Age | S |
| 76 | " | Male | CI: 0.87-1.52 | P | Age | L |
| 77 | " | Female | CI: 0.94-1.21 | P | Age | L |
| 78 | Roche et al. (2008) | Both | CI: 3.04-5.98 | P | Age, gender and marital status | ND |
| 79 | " | Both | CI: 4.46-12.08 | P | Age, gender and marital status | ND |
| 80 | Salonsalmi et al. (2009) | Female | CI: 1.12-1.54 | P | Age | S |
| 81 | " | Male | CI: 0.97-1.53 | P | Age | S |
| 82 | " | Female | CI: 1.34-2.10 | P | Age | S |
| 83 | " | Male | CI: 1.27-2.33 | P | '' | S |
| 84 | " | Female | CI: 1.23-1.43 | P | '' | S |
| 85 | " | Male | CI: 1.14-1.58 | P | '' | S |
| 86 | " | Female | CI: 1.06-1.60 | P | '' | L |
| 87 | " | Male | CI: 0.93-1.67 | P | '' | L |
| 88 | " | Female | CI: 1.24-2.17 | P | '' | L |
| 89 | " | Male | CI: 0.91-1.93 | P | '' | L |
| 90 | " | Female | CI: 1.09-1.32 | P | '' | L |
| 91 | " | Male | CI: 1.15-1.72 | P | '' | L |
| 92 | Norstrom & Moan (2009) | Male | *p* <0.05 | P | Unemployment rate | ND |
| 93 | " | Female | ns | P | '' | ND |
| 94 | Bacharach et al. (2010) | Both | ns | P | Gender, age, marital status, tenure, household income, average hours worked per week | ND |
| 95 | " | Both | *p* <0.01 | P | '' | ND |
| 96 | Balsa & French (2010) | Both | ns | N | Behavioral health and job characteristics | ND |
| 97 | " | Both | ns | P | '' | ND |
| 98 | " | Both | *p* <0.01 | P | '' | ND |
| 99 | Kirkham et al. (2015) | Both | *p* = 0.22 | P | Age group, region of residence, gender, work type, business unit, shift work, insurance plan, and exempt status | ND |
| 100 | Hensing et al. (2011) | Male | *p* = 0.703 | N | Age, Income, level of perceived symptoms | L |
| 101 | " | Female | *p* <.001 | P | '' | L |
| 102 | Edvardsen et al. (2015) | Both | *p* <.001 | P | No | ND |
| 103 | " | Both | *p* = 0.916 | N | No | ND |
| 104 | " | Both | *p* <.05 | P | No | ND |
| 105 | " | Both | *p* <.001 | P | No | ND |
| 106 | Lidwall & Marklund (2011) | Male | *p* <.001 | P | Calendar month | L |
| 107 | " | Female | *p* <.001 | P | Calendar month | L |
| 108 | Chakraborty & Subramanya (2013) | Both | *p* <.005 | P | No | ND |
| 109 | Schou et al. (2014) | Both | CI: 4.63-15.44 | P | No | ND |
| 110 | " | Both | CI: 6.00-22.10 | P | No | ND |
| 111 | Ervasti et al. (2018) | Both | CI: 1.16-1.66 | P | Age, socio-economic status, smoking, body mass index and physical and mental morbidity | ND |
| 112 | Ervasti et al. (2018) | Both | CI: 1.03-1.70 | P | Age, socio-economic status, smoking and body mass index | L |
| 113 | Torvik et al. (2016) | Both | CI: 0.9-1.6 | P | Gender, age, and parental education | L |
| 114 | Silva-Junior & Fischer (2014) | Both | CI: 1.18-85.63 | P | Age | L |
| 115 | Richmond et al. (2016) | Both | *p* <0.01 | P | Clinical mediators | ND |
| 116 | De Clercq et al. (2015) | Both | CI: 1.024-1.125 | P | Gender, age, education, occupational status, and health perception | L |
| 117 | Østby et al. (2016) | Both | CI: 2.42–4.71 | P | Gender | L |
| 118 | " | Both | CI: 1.58–3.72 | P | Gender | L |
| 119 | Morois et al. (2017) | Male | *p* <.01 | P | Age and socio-professional categories at hiring | ND |
| 120 | " | Female | *p* <.01 | P | '' | ND |
| 121 | " | Male | *p* <.01 | P | '' | S |
| 122 | " | Female | *p* <.01 | P | '' | S |
| 123 | " | Male | *p* <.01 | P | '' | L |
| 124 | " | Female | *p* <.01 | P | '' | L |
| 125 | Ervasti et al. (2018) | Male | CI: 1.23-2.23 | P | No | S |
| 126 | " | Female | CI: 0.99-1.30 | P | No | S |
| 127 | " | Male | CI: 1.19-2.21 | P | Smoking, BMI, and physical inactivity | S |
| 128 | " | Female | CI: 1.00-1.33 | P | Smoking, BMI, and physical inactivity | S |
| 129 | Salonsalmi et al. (2015) | Female | CI: 1.11 -1.75 | P | Age and marital status | S |
| 130 | " | Male | CI: 0.78–1.38 | P | '' | S |
| 131 | " | Female | CI: 1.17–1.43 | P | '' | S |
| 132 | " | Male | CI: 0.98–1.62 | P | '' | S |
| 133 | " | Female | CI: 0.94–1.64 | P | '' | L |
| 134 | " | Male | CI: 0.85–1.51 | P | '' | L |
| 135 | " | Female | CI: 0.97–1.23 | P | '' | L |
| 136 | " | Male | CI: 0.89–1.47 | P | '' | L |
| 137 | Araujo et al. (2017) | Both | CI: 0.13–11.64 | P | Gender, retirement age, income, sleep quality, and low back pain | ND |
| 138 | schou & Birkelund (2015) | Both | *p* < 0.001 | P | Age, gender, education, working size, income | ND |
| 139 | " | Both | *p* < 0.01 | P | '' | ND |
| 140 | " | Male | p < 0.01 | P | Age, education, working size, income | ND |
| 141 | " | Male | ns | P | '' | ND |
| 142 | " | Female | ns | P | '' | ND |
| 143 | " | Female | *p* < 0.01 | P | '' | ND |
| 144 | Kaila Kangas et al. (2018) | Both | CI: 0.98-1.35 | P | Age | L |
| 145 | " | Both | CI: 1.19-1.76 | P | Age | L |
| 146 | Jørgensen et al. (2017) | Female | *p* =0.20 | P | Age, cohabitation status, education, smoking, region | ND |
| 147 | " | Male | *p* = 0.02 | P | '' | ND |
| 148 | " | Female | *p* = 0.24 | P | '' | ND |
| 149 | " | Male | *p* = 0.89 | P | '' | ND |
| 150 | Jørgensen et al. (2019) | Both | CI: 1.17-1.43 | P | Age, gender, cohabitation status, educational level, mental illness and disorders, smoking behavior, geographic region, and labor market status | ND |
| 151 | " | Both | CI: 1.12-1.34 | P | '' | ND |
| 152 | Lund et al. (2019) | Both | *p* =0.147 | P | Age and education | S |
| 153 | " | Both | *p* = 0.991 | P | Age and education | L |
| 154 | Hambisa Mekonnen et al.  (2019) | Both | CI: 1.06–1.40 | P | Age and country of birth | S |
| 155 | Landberg et al. (2020) | Male | CI: 1.06–1.38 | P | '' | S |
| 156 | " | Female | CI: 1.11–1.59 | P | '' | S |
| 157 | " | Male | CI: 1.18–1.88 | P | '' | S |
| 158 | " | Female | CI: 1.05–2.74 | P | '' | L |
| 159 | " | Male | CI: 1.01–2.36 | P | '' | L |
| 160 | " | Female | CI: 1.27–3.58 | P | '' | L |
| 161 | " | Male | CI: 0.81–2.06 | P | '' | L |
| 162 | " | Female | *p* <.005 | P | Gender, age, marital status, education, and BMI | ND |
| ^a^ S = short-term; L = long-term; ND = number of absence days  ^b^ P = positive direction; N = negative direction | | | | | | |
