# Supplementary figures and images for: A systematic review and meta-analysis uncovering the relationship between alcohol consumption and sickness absence. When type of design, data, and sickness absence make a difference
Source: PLoS One. 2022 Jan 11;17(1):e0262458. doi: 10.1371/journal.pone.0262458 (PMC8752011; doi:10.1371/journal.pone.0262458)

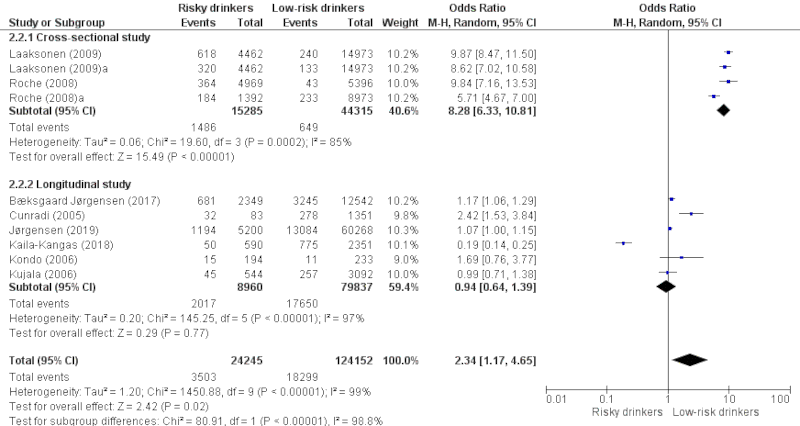

Supplement: S1 Fig — (TIF) [file pone.0262458.s004.tif]

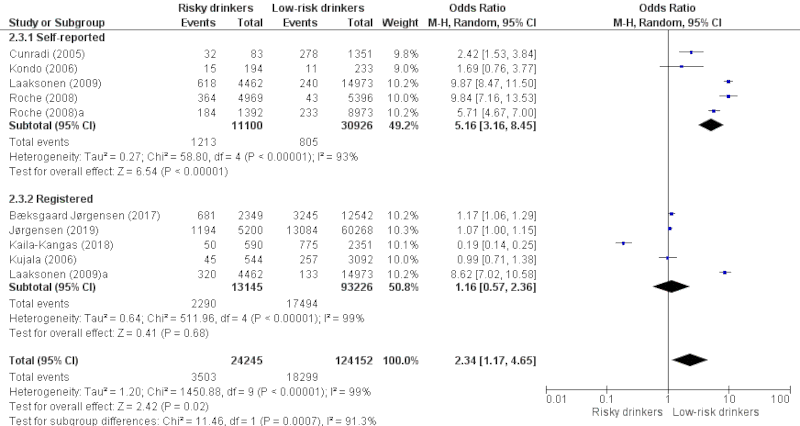

Supplement: S2 Fig — (TIF) [file pone.0262458.s005.tif]

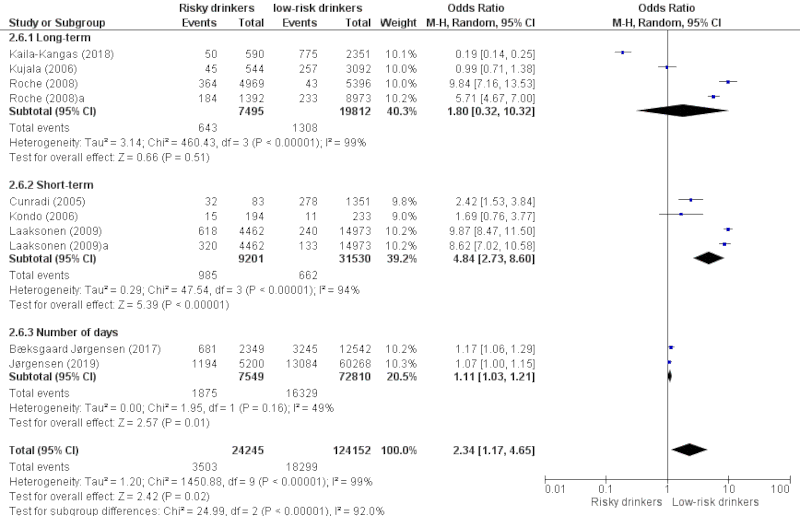

Supplement: S3 Fig — (TIF) [file pone.0262458.s006.tif]

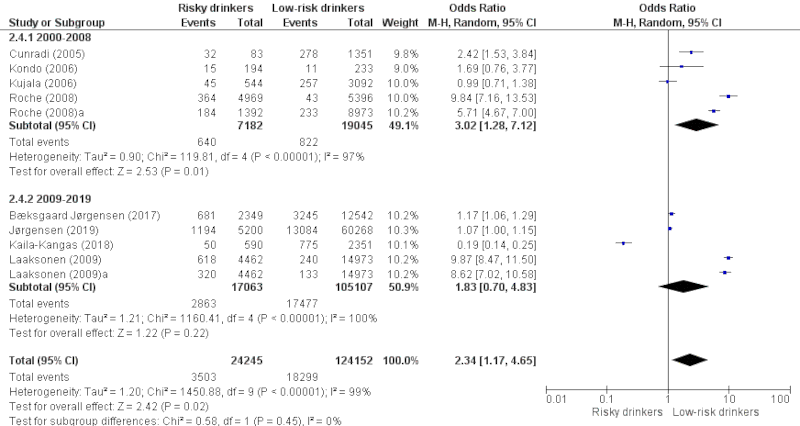

Supplement: S4 Fig — (TIF) [file pone.0262458.s007.tif]

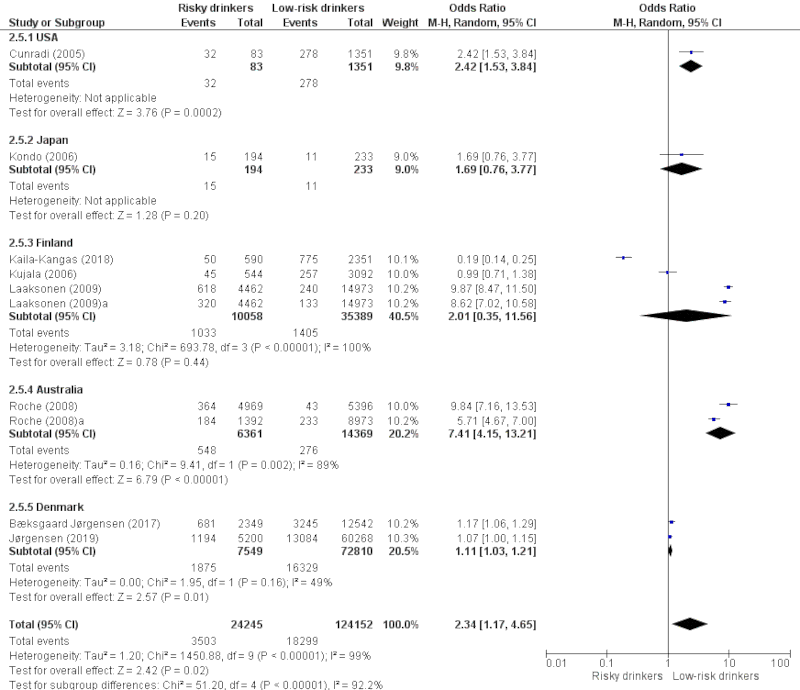

Supplement: S5 Fig — (TIF) [file pone.0262458.s008.tif]
